# MANET 3.0: Hierarchy and modularity in evolving metabolic networks

**DOI:** 10.1371/journal.pone.0224201

**Published:** 2019-10-24

**Authors:** Fizza Mughal, Gustavo Caetano-Anollés

**Affiliations:** 1 Illinois Informatics Institute, University of Illinois at Urbana-Champaign, Urbana, Illinois, United States of America; 2 Evolutionary Bioinformatics Laboratory, Department of Crop Sciences, University of Illinois at Urbana-Champaign, Urbana, Illinois, United States of America; ICAR - Indian Institute of Horticultural Research (IIHR), INDIA

## Abstract

Enzyme recruitment is a fundamental evolutionary driver of modern metabolism. We see evidence of recruitment at work in the metabolic Molecular Ancestry Networks (MANET) database, an online resource that integrates data from KEGG, SCOP and structural phylogenomic reconstruction. The database, which was introduced in 2006, traces the deep history of the structural domains of enzymes in metabolic pathways. Here we release version 3.0 of MANET, which updates data from KEGG and SCOP, links enzyme and PDB information with PDBsum, and traces evolutionary information of domains defined at fold family level of SCOP classification in metabolic subnetwork diagrams. Compared to SCOP folds used in the previous versions, fold families are cohesive units of functional similarity that are highly conserved at sequence level and offer a 10-fold increase of data entries. We surveyed enzymatic, functional and catalytic site distributions among superkingdoms showing that ancient enzymatic innovations followed a biphasic temporal pattern of diversification typical of module innovation. We grouped enzymatic activities of MANET into a hierarchical system of subnetworks and mesonetworks matching KEGG classification. The evolutionary growth of these modules of metabolic activity was studied using bipartite networks and their one-mode projections at enzyme, subnetwork and mesonetwork levels of organization. Evolving metabolic networks revealed patterns of enzyme sharing that transcended mesonetwork boundaries and supported the patchwork model of metabolic evolution. We also explored the scale-freeness, randomness and small-world properties of evolving networks as possible organizing principles of network growth and diversification. The network structure shows an increase in hierarchical modularity and scale-free behavior as metabolic networks unfold in evolutionary time. Remarkably, this evolutionary constraint on structure was stronger at lower levels of metabolic organization. Evolving metabolic structure reveals a *‘principle of granularity’*, an evolutionary increase of the cohesiveness of lower-level parts of a hierarchical system. MANET is available at http://manet.illinois.edu.

## Introduction

Enzyme recruitment is believed to play a central role in metabolic evolution [1]. Under this evolutionary scenario, genes spread by duplication in genomes while variants of the encoded enzymes, which were originally multifunctional, are coopted into different metabolic pathways. These variants are consequently tailored by specialization to meet the specific functional demands of those pathways. Here we delve deeper into the origins and evolution of modern metabolism, exploring if the recruitment of enzymes in metabolic networks is currently at work. We also study the emergent properties of the structure of evolving networks.

There is significant evidence supporting the early evolutionary role of enzyme recruitment. Phylogenomic analysis revealed that some of the early protein structural domains comprise 3-dimensional fold structures that are widely present in metabolic enzymes [2,3]. These structures were part of proteins responsible for nucleotide synthesis, indicating ancient domains were instrumental in providing the molecular functions for a developing primordial RNA world [4]. Collectively, findings suggest a “metabolism-first” model of protein evolution, which finds its basis at a strongly linked evolutionary level of protein structure, the fold family [5].

The metabolic Molecular Ancestry Network (MANET) database is a useful resource to investigate the evolution of protein domains in modern metabolism [6]. The evolutionary age of the domains is directly derived from phylogenetic trees reconstructed from a census of protein domain structures in genomes [2,7–9]. The age of these domain structures is then “painted” onto the enzymes of metabolic pathways defined by the Kyoto Encyclopedia of Genes and Genomes (KEGG) [10]. The metabolic MANET database has facilitated the retrodiction of ancient enzyme functions describing “metaconsensus enzymes” [11]. In addition, it has bolstered support for the existence of protein evolution prior to the inception of the modern translation system, challenging the traditional view that RNA molecules appeared before proteins [12,13]. Most of the ancestral fold domains identified are at the heart of metabolism [4]. By using “subnetwork wheels”, ‘Purine metabolism’ and ‘Pyrimidine metabolism’ were found to represent the most ancestral subnetworks of metabolism [4].

There is however a need to update metabolic MANET, which was originally designed to trace domain evolution at fold level of the Structural Classification of Proteins (SCOP), one of the two gold standards of protein taxonomy [6]. In contrast with SCOP folds and the embedded fold superfamilies, the evolutionary relatedness of fold families can be derived directly from protein sequences, sometimes without invoking the structure and function of the molecules [9]. In addition, fold families reflect a clear embodiment of domain functionality, which helps in the assignment of features of sequence, structure and function to domains when these are traced along the evolutionary timeline of protein history. Thus, fold families reap the benefits of protein structure by encompassing deep evolutionary views and the benefits of protein sequence by enabling unequivocal assignments of molecular functions [9]. Here we update metabolic MANET by tracing fold family history in metabolic networks. This solves the potential problems of the relatively loose link that exits between folds and evolution and offer better ways to explore enzymatic recruitment in metabolic networks. The benefit of using fold families has been recently highlighted by the evolutionary study of purine metabolism [5], which uncovered the origin of metabolism by gradual replacement of biotic chemistries with catalytic proteinaceous counterparts. The study revealed strong underlying phylogenetic signal in metabolic enzymes. The new release of MANET also links to data in the PDBsum database [14,15], bridging enzymatic and structural information. Using MANET, we now show that network analyses of the wiring diagrams of metabolic organization reveal evolutionary patterns in the structure of metabolic networks and ongoing metabolic growth through pervasive enzymatic recruitment.

## Results

### Dissecting enzyme recruitment and metabolic network structure

**Fig 1:**
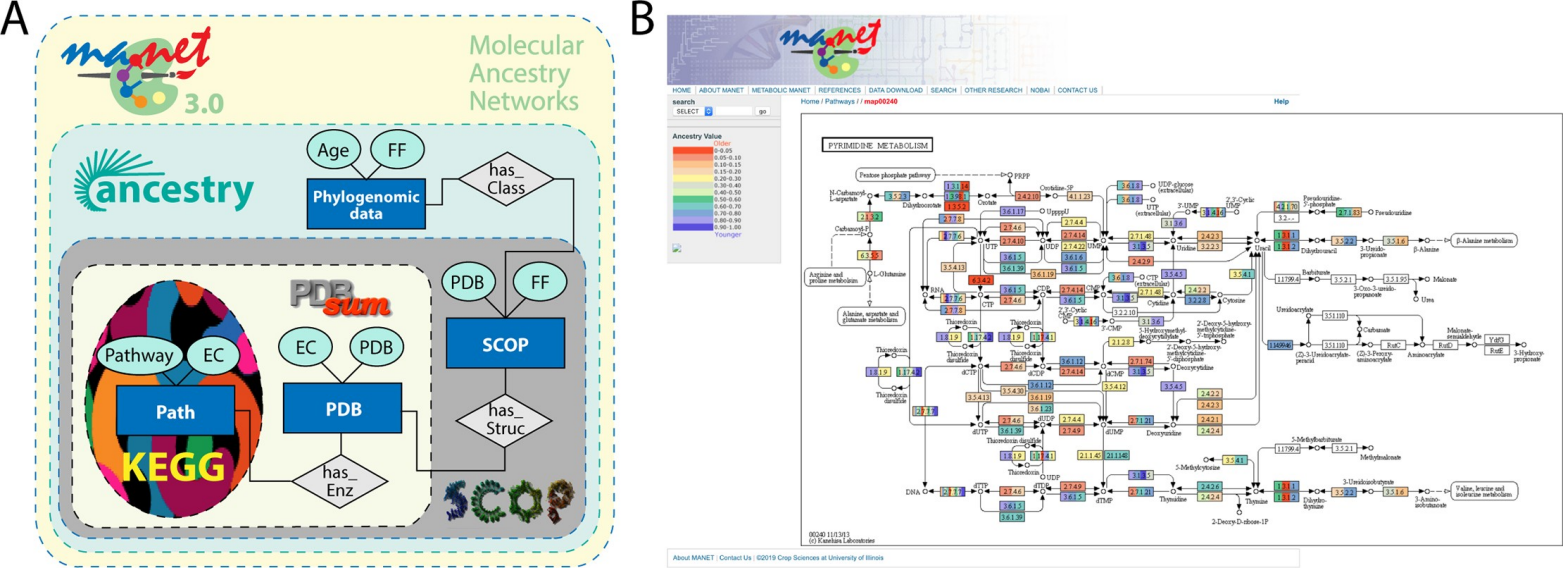
Metabolic MANET 3.0. (A) Entity relationship model of the updated version of metabolic MANET linking ancestries, SCOP, PDBsum and KEGG. (B) Screenshot of a representative subnetwork diagram describing the ‘Pyrimidine metabolism’ subnetwork of MANET. A color scale is used to assign binned ancestry values to enzyme nodes named with EC numbers.

Release 3.0 of the metabolic MANET database (February 2019) now provides an evolutionary visualization of metabolic pathways at fold family level of SCOP. First, structural domains are mapped onto PDB entries using SCOP, followed by mapping of PDB entries onto EC numbers via PDBsum (Fig 1A). The evolution of metabolic networks is made explicit by color-coding the age of families of structural domains in the enzymes of the metabolic pathway diagrams of KEGG. The ages of these domain structures are denoted by *‘*ancestry’ values, measures of node distance (*nd*) derived directly from phylogenomic trees that range from 0 (the most ancient domains) to 1 (the most recent). Enzymatic activities showing multiple colorings (ancestry values) result from taxonomical differences in domain makeup or simply the existence of multiple domains in enzymes. KEGG is a computational view of the wiring diagrams of biochemical reactions and molecular interactions that make up the metabolic system. These diagrams group enzymes responsible for functionally related metabolic pathways into reference network maps, which we call ‘subnetworks’ [6]. Subnetworks that have similar functional capacities are further grouped into ‘mesonetworks’, which can be considered upper level categorizations of the metabolic network [6]. For example, the ‘Purine metabolism’ and ‘Pyrimidine metabolism’ subnetworks, which are mainly responsible for the synthesis, degradation and salvage of purine and pyrimidine metabolites (and related compounds), respectively, are members of the Nucleotide metabolism (NUC) mesonetwork. Fig 1B illustrates the evolutionary mappings with the color tracings of the ‘Pyrimidine metabolism’ subnetwork of MANET.

The functionality of MANET’s search engine makes it searchable in terms of enzymes, PDB entries, SCOP domains and subnetworks. The output is presented in a tabulated form including the domain and corresponding *nd* values of individual enzymes. Compared to its predecessor, MANET 3.0 shows a 10-fold increase of data entries (Table 1), with 97.4% subnetwork coverage (S1 Table) and increased painting efficiency (S1 Fig and S2 Table).

**Table 1:** MANET 3.0 database statistics.

Database entities	Version 3.0	Version 2.0 [6]
Total entries	240,348	23,217
Metabolic pathway enzymes	1,925 (out of 2,867 KEGG enzymes)	1,255 (out of 2,015 KEGG enzymes)
PDB entries	21,980	6,552
SCOP entries	1,610 (out of 3,513 families)	784 (out of 2,493 families/887 folds)
KEGG metabolic pathways in MANET that have associated enzymes	148 (out of 151 pathways)	132 (out of 137 pathways)
Enzymes with crystallographic information	919	758
HMM assignments at e-value = 0.0001	1,006	584
Average painting coverage	72.68%	71.8%

**Fig 2:**
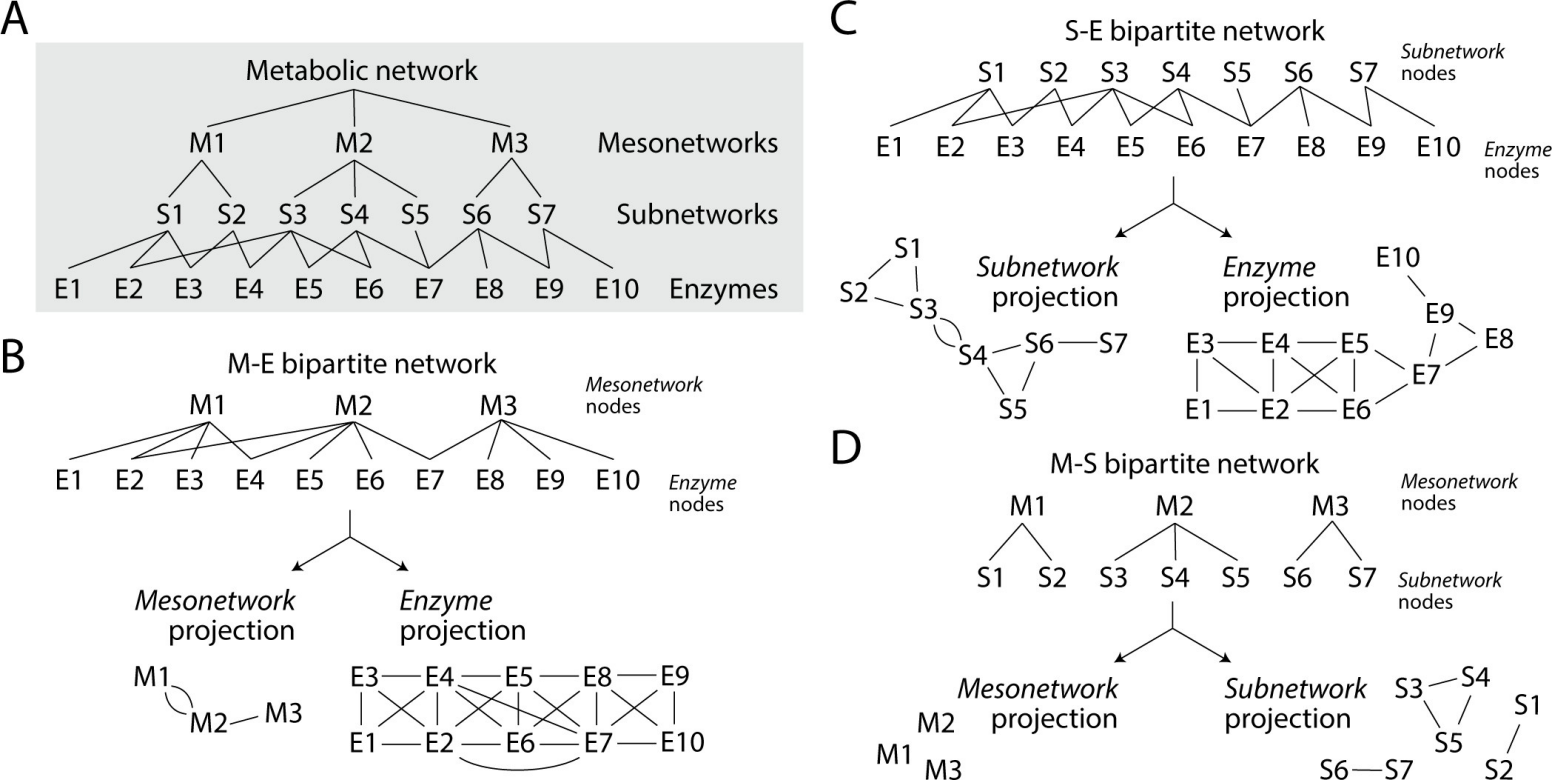
A network view of metabolism. (A) The enzymatic activities (E) of the metabolic network can be dissected into a hierarchical system of subnetworks (S) and mesonetworks (M), which act as modules of metabolic activity. (B) A bipartite network describing the relationship between mesonetworks and enzymes can be dissected into its two one-mode projections, one describing how enzymes link mesonetworks to each other, the other describing how mesonetworks link enzymes to each other. (C) A bipartite network of subnetworks and enzymes can be dissected into its two one-mode projections, one describing how enzymes link subnetworks to each other, the other describing how subnetworks link enzymes to each other. (D) A bipartite network of mesonetworks and subnetworks can be dissected into its two one-mode projections, one describing how subnetworks link mesonetworks to each other, the other describing how mesonetworks link subnetworks to each other.

Mapping the age of enzymes onto mesonetworks and corresponding subnetworks in MANET uncovered evolutionary patterns of enzymatic recruitment. Fig 2A shows a diagram describing how enzymes, subnetworks and mesonetworks form a hierarchical system of functional metabolic modules defined at different levels of organization, which are tightly wired to each other through the common activities of enzymes. To better study the evolving network structure of this hierarchy of modules, we generated undirected bipartite networks that link mesonetwork and subnetwork levels of KEGG pathway classification to enzymes (Fig 2B and 2C). We then focused on one-mode projections of these bipartite networks to visualize how the individual levels of network organization contribute to overall network structure. Note that three possible bipartite networks arise from the hierarchy of enzymes, subnetworks and mesonetworks (Fig 2B, 2C and 2D). Networks describing the relationship of neighboring hierarchical levels are linked more densely the lower in the hierarchy. Non-neighbor relationships also result in density increase. We do not study the bipartite network that links mesonetworks to subnetworks because it provides little information (Fig 2D).

### A bipartite network of mesonetworks and enzymes

**Fig 3:**
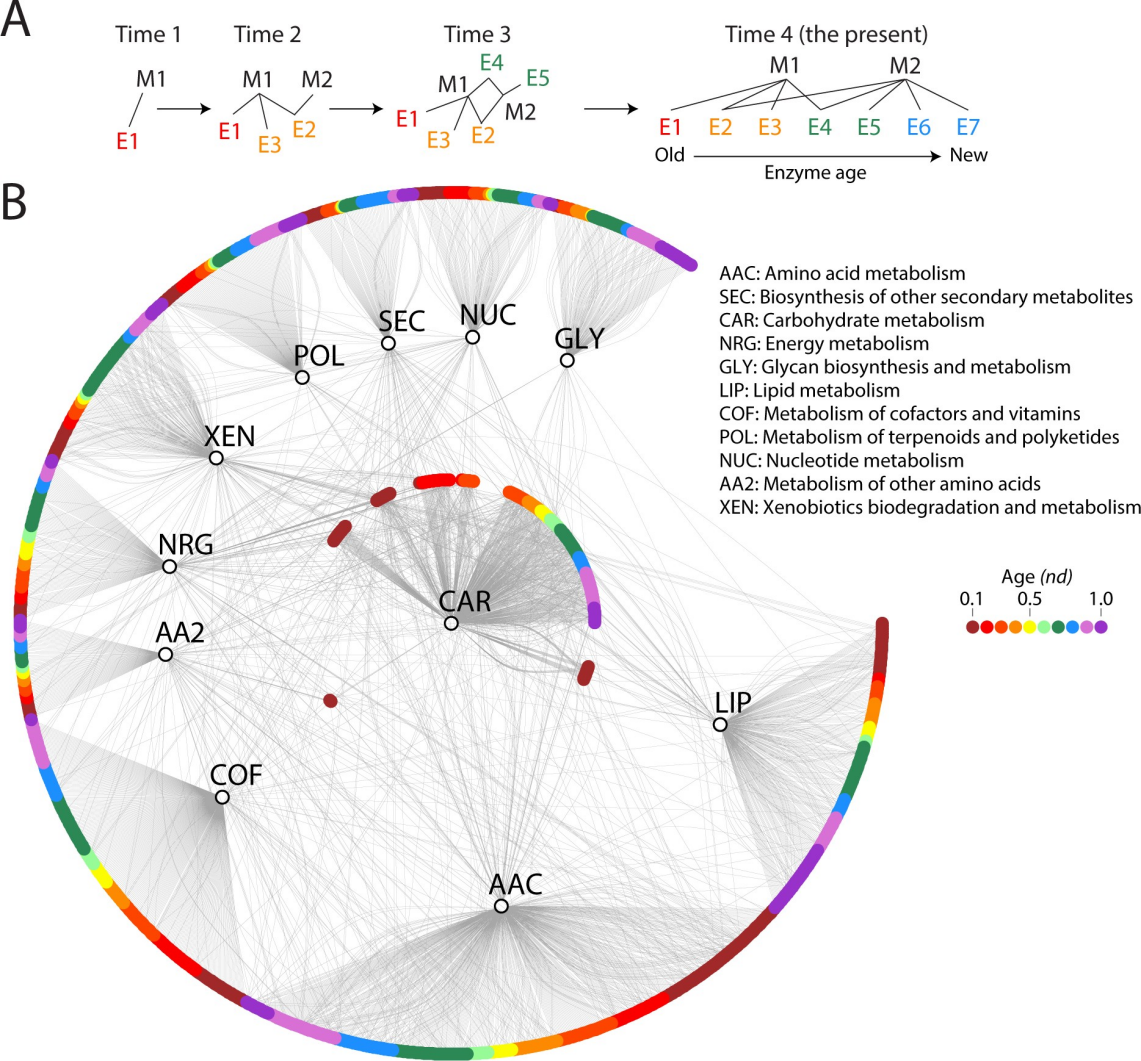
Evolution of the mesonetwork-enzyme bipartite network. (A) Tracing enzyme ages on the bipartite networks, facilitates studying patterns of sharing and show the evolution of networks in time (B) A bipartite graph of mesonetworks and enzymes (*nd* = 1.0) showing enzymes by *nd* distribution on a scale of red to violet representing ancestral to recent fold family domain assignments. Mesonetworks are shown as vertices in black while colored nodes denote enzymes.

We first constructed an undirected bipartite network linking the 11 mesonetworks to their corresponding enzymes, which were indexed with the evolutionary age of their structural domains (Fig 3). The mesonetwork nodes were connected to each other through common enzyme nodes. The evolution of connections among the mesonetworks were unfolded at each step of the timeline by tracing the age of enzymes on the bipartite network (Fig 3A). Carbohydrate (CAR) and Amino Acid (AAC) mesonetworks showed the largest number of enzyme connections in the bipartite network while the rest exhibited moderate connectivity (Fig 3B). In these analyses, the age of an enzyme was considered to be the age of its second oldest structural domain, if multiple domains were present in the enzyme. Applying the age criterion to the bipartite network permitted to build a series of bipartite networks that were evolving in time. Note that while the age of a cooption is necessarily determined by the age of the youngest domain of an enzyme, i.e. the age of the acceptor of older domains, the actual age of the enzyme is determined by the oldest component domain, i.e. the oldest donor in the cooption, without which the cooption would not be possible. In this regard, the global evolutionary patterns we here report for the bipartite networks were consistent to those obtained when the age of a multidomain enzyme was considered to be the age of its oldest structural domain [16]. These additional results are provided in S4–S6 Figs, confirming that the strategy to assign ages to multidomain enzymes does not affect the overall conclusions of our study.

**Fig 4:**
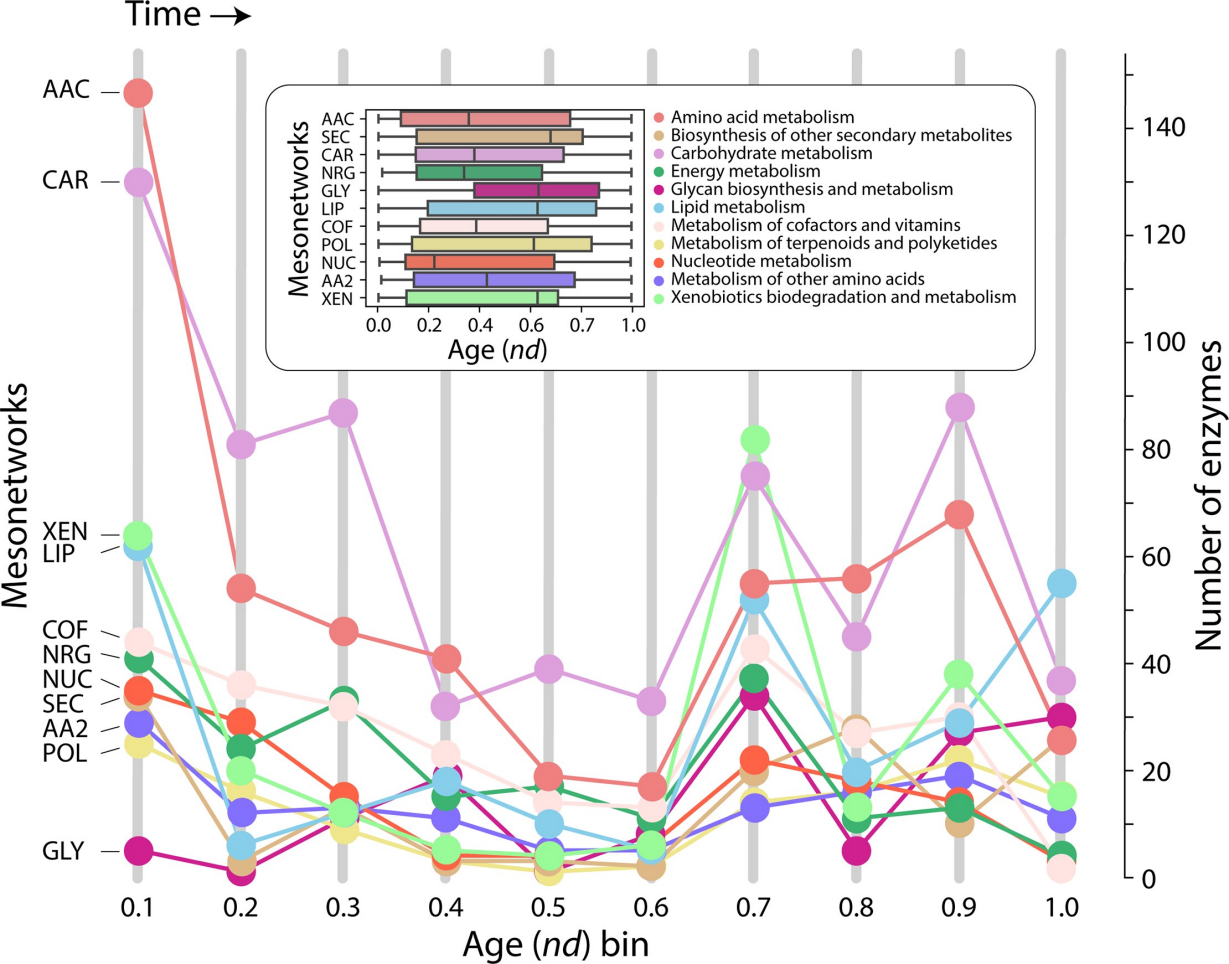
Run chart of enzymes in mesonetworks appearing in each *nd* era. Eras are defined as *nd* bins of ages; the first *nd* bin includes enzymes appearing between *nd* = 0 and *nd* = 0.1. The inset describes the distribution of enzymes along the evolutionary timeline.

**Fig 5:**
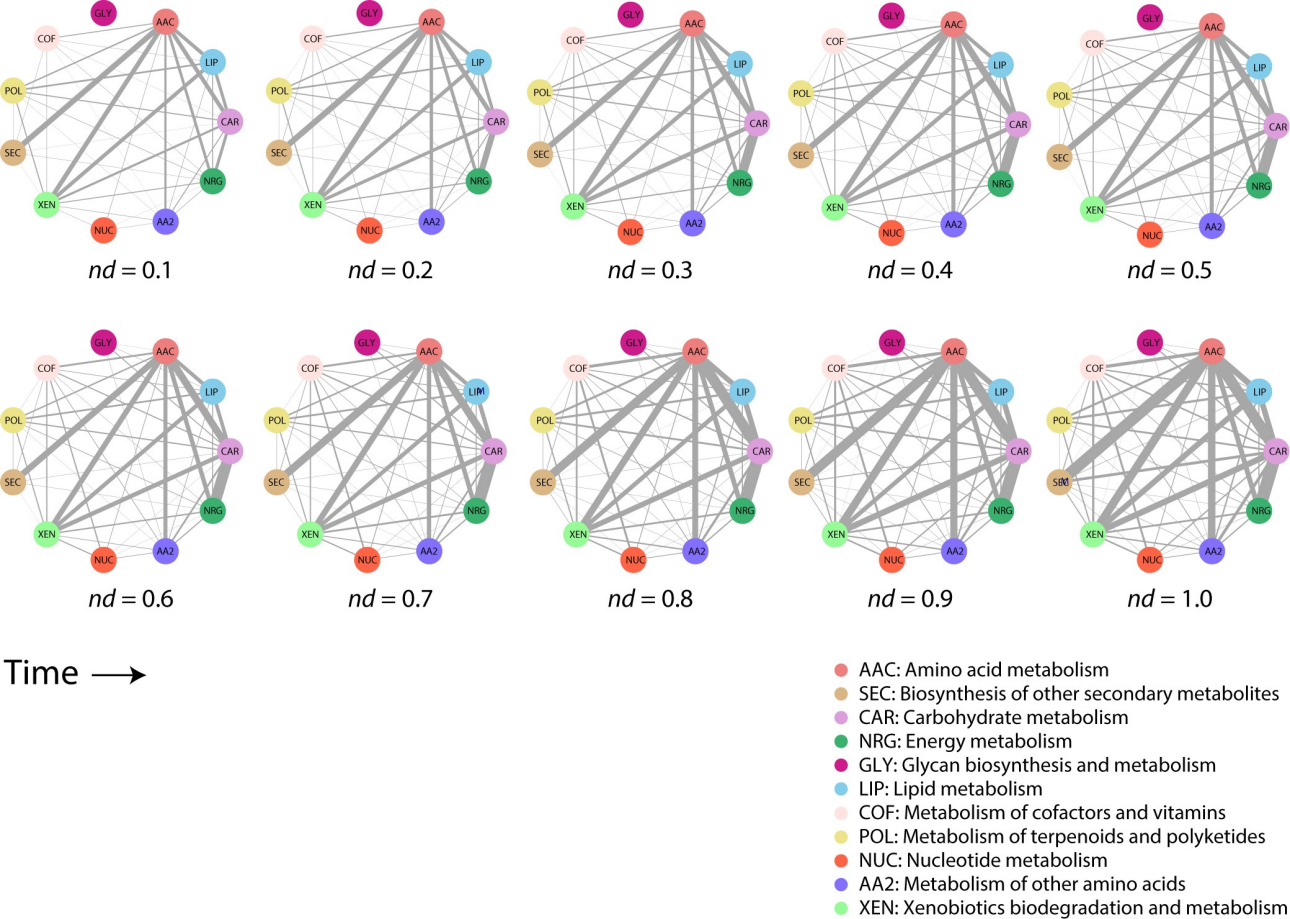
Connectivity patterns among mesonetworks at different stages of the evolutionary timeline. Mesonetworks are represented by vertices while edge thickness shows the number of enzymes shared. AAC, Amino acid metabolism; SEC, Biosynthesis of other secondary metabolites; CAR, Carbohydrate metabolism; NRG, Energy metabolism; GLY, Glycan biosynthesis and metabolism; LIP, Lipid metabolism; COF, Metabolism of cofactors and vitamins; POL, Metabolism of terpenoids and polyketides; NUC, Nucleotide metabolism; AA2, Metabolism of other amino acids; XEN, Xenobiotics biodegradation and metabolism.

When studying evolving networks, we identified two distinct peaks in the appearance of domains used by the enzymes along the timeline, at age *nd* = 0.0–0.1 and later at *nd* = 0.6–0.7, with a period of gradual decline in between (Fig 4). This biphasic behavior is typical of domain innovation [17]. Second, a one-mode network projection for mesonetworks (Fig 5) extracted from the bipartite network (Fig 3), which follows the criteria described in Fig 2B, highlights the dominant sharing patterns unfolded by the Amino acid (AAC) mesonetwork earlier in the timeline (*nd* = 0.1–0.2). At *nd* = 0.2, the carbohydrate and energy metabolism mesonetworks established a strong sharing bond, which strengthened throughout the timeline. The Glycan biosynthesis and metabolism (GLY) mesonetwork initiates sharing of enzymes at *nd* = 0.3, starting with the Carbohydrate (CAR) mesonetwork and then proceeding to share enzymes with the Other amino acids (AA2), Amino acid (AAC), Lipid (LIP), Secondary metabolites (SEC), Cofactors and vitamin (COF) and Xenobiotics (XEN) mesonetworks at *nd* values of 0.4, 0.6, and 0.9. Interestingly, the Glycan biosynthesis and metabolism (GLY) mesonetwork did not participate in sharing enzymes with Energy (NRG), Terpenoids and polyketides (POL) and Nucleotide metabolism (NUC) mesonetworks.

### A bipartite network of subnetworks and enzymes

In order to explore the processes of enzyme recruitment, we also constructed undirected bipartite networks at the subnetwork level, which consisted of two disjoint sets of nodes, one describing subnetworks and the other describing enzymes (following entity and relationship criteria defined in Fig 2C). In these networks, each metabolic subnetwork node connects to each other through a common enzyme node shared by subnetworks. We also decomposed each bipartite graph into its two one-mode projections, a *‘subnetwork’* network and an *‘enzyme’* network. We explored the scale-freeness, randomness and small-world properties of the bipartite graph and those of the one-mode projections to uncover patterns in the evolution of network structure.

**Fig 6:**
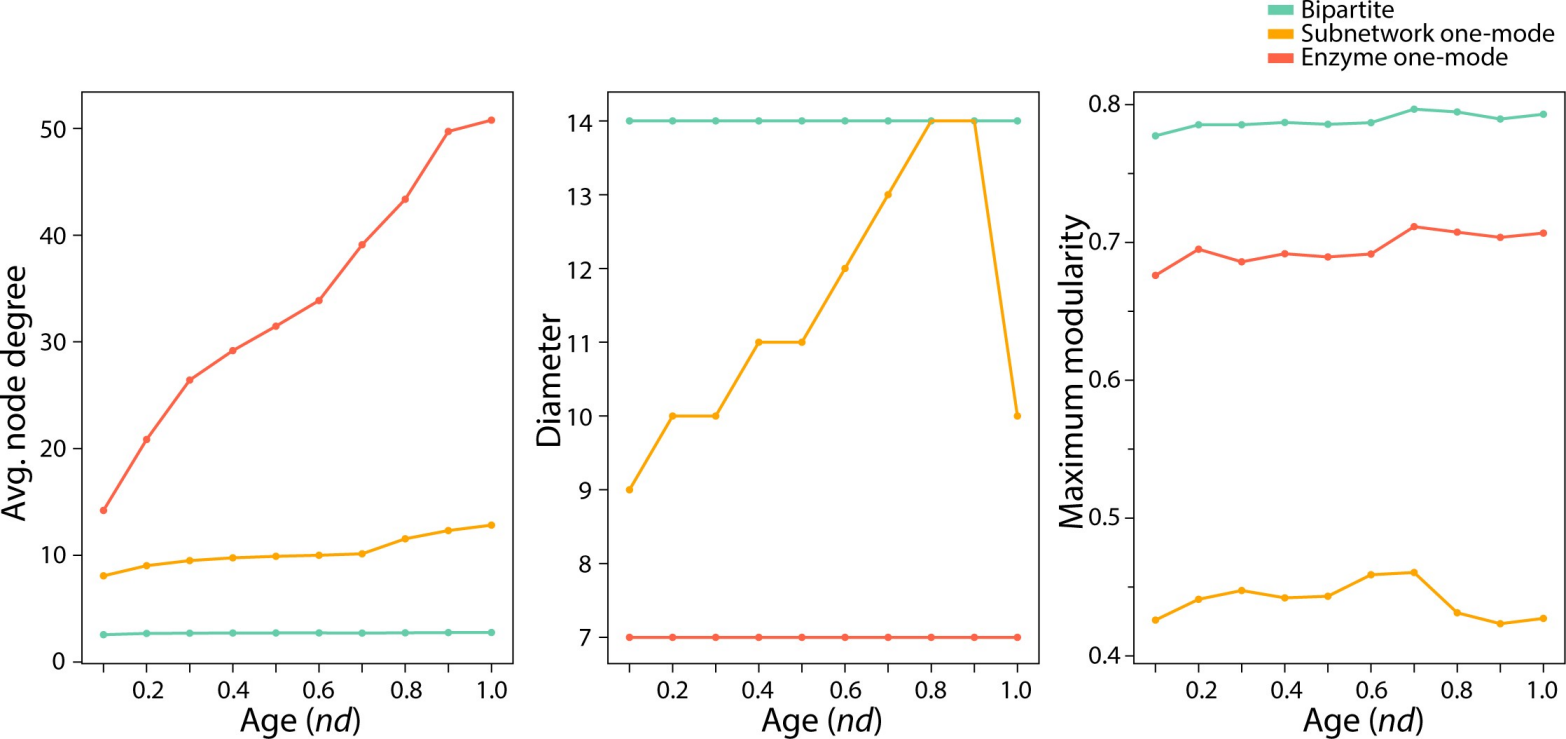
Average node degrees (average number of links), diameter and maximum modularity scores for each type of network (largest connected component) at each time point (0.1 *nd* interval). Network sizes (total number of nodes and nodes in the largest connected component) are given in S3 Fig.

**Fig 7:**
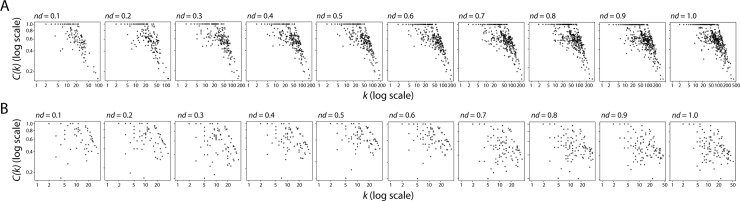
Log-log plot of *C(k) vs k* for the one-mode enzyme (A) and subnetwork (B) projections at *nd* value intervals of 0.1.

Kolmogorov-Smirnov tests for each of these three graphs at all *nd* levels showed consistent patterns of scale-freeness in the bipartite graphs but their absence in derived one-mode projections. Log-log plots were created using a fitting function [18] that applies the Kolmogorov-Smirnov test to the data (S2 Fig). A good fit is indicated by lower values of the Kolmogorov-Smirnov test statistic (KS.stat) and higher p-values. The hypothesis that a distribution follows a power law is rejected when the p-value is less than 0.05. The log likelihood (logLik) for the fitted parameters is computed and its value ranges from 0 to 1, with 0 indicating that the parameters are a better fit for the data set being analyzed. In order for the networks to be truly scale-free, the power law exponent value (alpha) must lie between 2 and 3. In accordance with these values of power law exponent, our subnetwork-enzyme bipartite graph exhibited power law distribution tendencies at all stages of the timeline, with acceptable Kolmogorov-Smirnov test statistic and p-values (Table 2). In contrast, analyses of one-mode network projections rejected power law behavior and showed networks lacked significant scale-freeness (Table 2). The Bartels’ rank test of randomness [19] for each graph revealed significant randomicity when compared to a corresponding Erdős–Rényi (ER) random graphs, but no significant trends across the timeline. In all cases, the null hypothesis that network data had been drawn from a random distribution was rejected for the three types of graphs (Table 3). In terms of small-world behavior, the bipartite graphs retained constant diameter throughout the timeline (Fig 6), in accordance with previous studies of metabolic networks [20]. However, while the enzyme one-mode projection retained the constant diameter property, the subnetwork one-mode projection increased diameter with time, reaching a peak at *nd* = 0.8–0.9 (Fig 6A). The maximum modularity scores recorded for the bipartite graph at all *nd* values also corresponded to behavior observed in metabolic networks [21]. They increased steadily with evolutionary time (Fig 6). Similar tendencies were clear for one-mode projections, with the exception that the modularity scores of the subnetwork one-mode projections decreased after peaking at *nd* = 0.6–0.7. Note however that overall modularity levels of the enzyme and subnetwork one-mode projections decreased ~10% and ~50%, respectively, relative to the bipartite network. This is expected since the bipartite network describes the cohesiveness between two levels of metabolic organization, while the one-mode projections describe the individual contribution of the levels to modular behavior. Finally, hierarchy in networks can be enumerated by the relationship of the clustering coefficient of a node with *k* edges, *C(k)*, which must follow a scaling law *C*(*k*)~*k*^−1^ [21]. We computed the *C(k)* function for the one-mode projections at each *nd* value (Fig 7). The *C(k)* power law scaling relationship increased in strength with evolutionary time (Fig 7), but was significantly weaker for the subnetwork one-mode projection. Analysis of networks annotated with the age of the most ancestral enzyme domain in a multi-domain enzyme revealed the same topological behaviors and evolutionary trends observed when networks were annotated with the age of the second oldest domain (S7, S8 and S13 Figs, S4 and S5 Tables). Since the clustering coefficient cannot be trivially calculated for a bipartite network, we performed calculations to assess small-world behavior only for the one-mode projection graphs, which we now discuss.

**Table 2:** Parameters for the power law fitting function in R for the bipartite network corresponding to the plots in S2 Fig at different *nd* values. alpha: exponent for the fitted power law distribution, xmin: lower bound for the power law fitting, logLik: log-likelihood of fitted parameters, KS.stat: test statistic for the Kolmogorov-Smirnov test between fitted and sample distribution and KS.p: p-value for the Kolmogorov-Smirnov test between fitted and sample distribution. The null hypothesis is that the original data has been drawn from a fitted power-law distribution. p-values less than 0.05 imply that the null hypothesis is rejected).

Network	*nd* Value	alpha	xmin	logLik	KS.stat	KS.p
**Bipartite**	0.1	2.036126	1	-759.4186	0.049686	0.1849861
	0.2	2.050221	1	-1041.634	0.0332531	0.4482009
	0.3	2.09732	1	-1294.618	0.0190888	0.9069918
	0.4	2.110523	1	-1455.256	0.0199788	0.8205487
	0.5	2.127836	1	-1550.445	0.0197397	0.7930782
	0.6	2.140443	1	-1641.388	0.0199515	0.7449955
	0.7	2.18352	1	-2031.524	0.0209772	0.5249036
	0.8	2.192613	1	-2244.833	0.0220464	0.3914344
	0.9	2.212848	1	-2525.648	0.0223349	0.294738
	1	2.222591	1	-2707.187	0.0227664	0.2328535
**Subnetwork one-mode**	0.1	1.428476	1	-320.807	0.2497108	0.0000388
	0.2	1.770404	1	-400.6262	0.4805927	0
	0.3	1.703825	1	-428.3908	0.4520308	0
	0.4	1.665948	1	-436.6208	0.4466223	0
	0.5	1.403904	1	-423.5062	0.2688292	0.0000003
	0.6	1.670431	1	-464.616	0.455313	0
	0.7	1.394778	1	-499.3063	0.294384	0
	0.8	1.376154	1	-525.1268	0.2953485	0
	0.9	1.370872	1	-544.0731	0.2951147	0
	1	1.364435	1	-565.1191	0.3160804	0
**Enzyme** **one-mode**	0.1	1.722109	1	-1699.593	0.5721901	0
	0.2	2	1	-3218.503	0.763262	0
	0.3	2	1	-4700.475	0.7858296	0
	0.4	2	1	-5670.544	0.811858	0
	0.5	2	1	-6374.768	0.8179033	0
	0.6	2	1	-7037.294	0.819453	0
	0.7	2	1	-9623.439	0.8377243	0
	0.8	2	1	-11144.61	0.845592	0
	0.9	2	1	-13429.85	0.8642292	0
	1	2	1	-14726.7	0.8716912	0

**Table 3:** Results of the Bartels’ test for randomness performed on each type of network at each time-point (0.1 *nd* interval) as well as for an equivalent Erdős–Rényi (ER) random graph. The null hypothesis is that the underlying data has been drawn from a random distribution. p-values less than 0.05 indicate that the null hypothesis is rejected.

Network	*nd* Value	Bartels' Statistic	Bartels' p-value	ER Bartels' Statistic	ER Bartels’ p-value
**Bipartite**	0.1	-9.26579	2.81E-22	0.19949	0.8421185
	0.2	-11.41278	2.82E-33	-0.0718005	0.9428243
	0.3	-13.01884	8.64E-43	1.433994	0.1516841
	0.4	-13.73945	2.38E-47	1.093474	0.2744027
	0.5	-14.10472	1.16E-49	0.7152102	0.474733
	0.6	-14.06547	4.97E-49	0.6531845	0.5138707
	0.7	-14.76641	3.97E-53	-1.240499	0.2149025
	0.8	-15.4329	8.90E-58	-1.035672	0.3004946
	0.9	-15.70583	2.50E-59	0.08607462	0.9314338
	1	-16.4897	2.66E-65	1.266439	0.2054321
**Subnetwork one-mode**	0.1	-2.395901	1.59E-02	-0.3585369	0.7217074
	0.2	-3.198736	1.15E-03	-1.789701	0.07336275
	0.3	-4.588882	1.82E-06	0.6698046	0.5051637
	0.4	-4.505029	2.93E-06	-0.0746503	0.9408455
	0.5	-4.612485	1.60E-06	0.08810647	0.9302074
	0.6	-4.077341	2.75E-05	-0.1756853	0.8613286
	0.7	-4.8855	3.81E-07	-0.7944839	0.4287919
	0.8	-4.659426	1.42E-06	-0.3000515	0.7652778
	0.9	-4.774271	7.34E-07	-1.059524	0.2909151
	1	-4.646165	1.55E-06	0.01763957	0.9859971
**Enzyme one-mode**	0.1	-9.427427	9.11E-24	-0.0042724	0.9965979
	0.2	-9.716918	3.07E-24	0.5071284	0.6125028
	0.3	-11.17966	1.64E-31	3.444245	0.00055136
	0.4	-11.8135	7.06E-35	-1.607051	0.1080852
	0.5	-11.91661	3.04E-35	0.712586	0.4763889
	0.6	-12.10103	3.61E-36	-0.519697	0.6035144
	0.7	-12.6368	8.49E-39	-1.865451	0.06209498
	0.8	-14.16609	1.15E-48	1.044054	0.2966115
	0.9	-15.06327	1.07E-54	-0.3197554	0.7492529
	1	-16.41173	5.20E-65	-0.0146644	0.9883044

### Subnetwork one-mode projections

**Fig 8:**
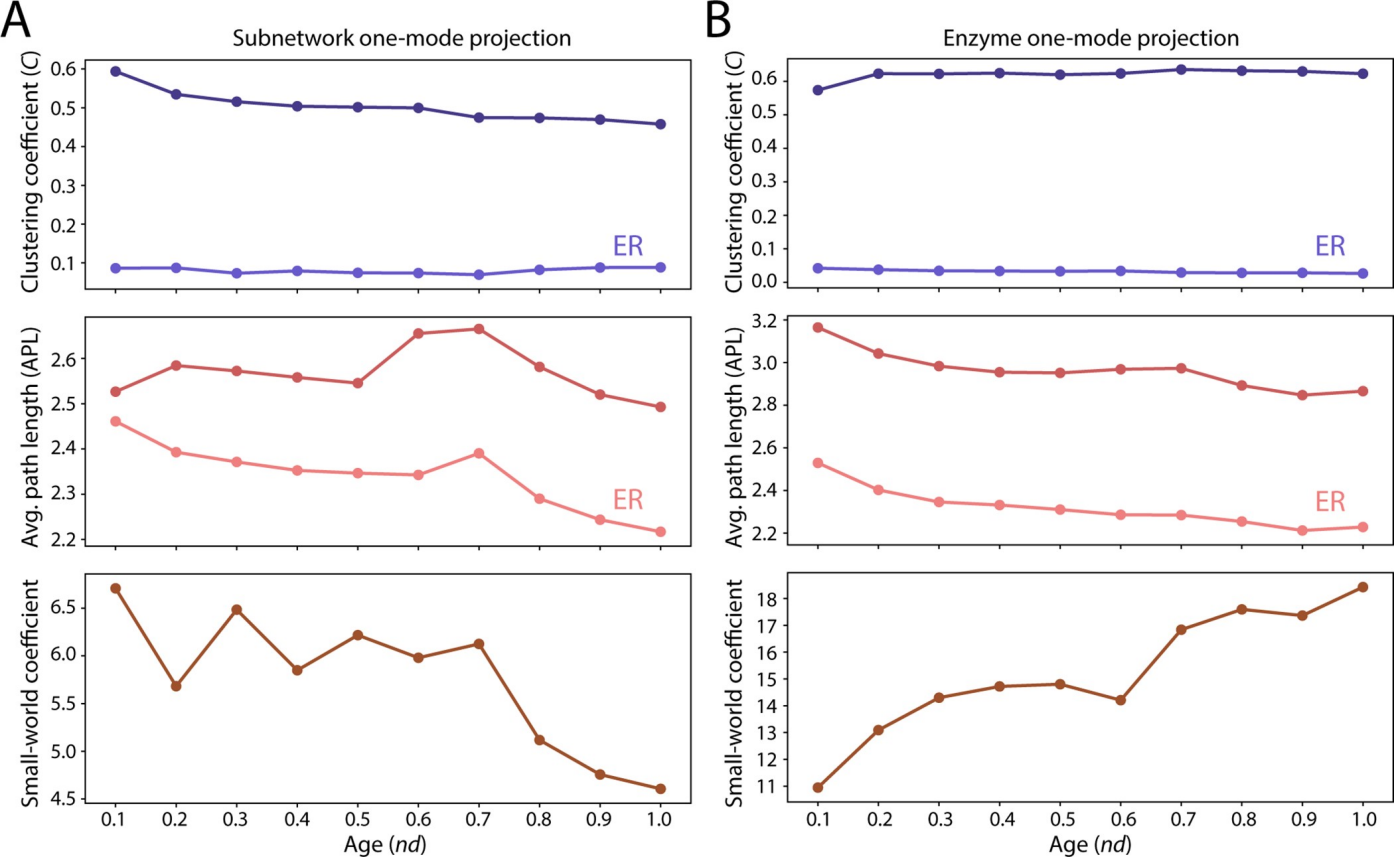
Testing for small-world behavior in the subnetwork and enzyme one-mode networks. (A) Comparison of clustering coefficient and average path length of the subnetwork one-mode network to that of an Erdős–Rényi (ER) network. The small-world coefficients decrease with the passage of time. (B) Comparison of clustering coefficient and average path length of the enzyme one-mode network to that of an Erdős–Rényi (ER) network. The resulting small-world coefficient increase along the evolutionary timeline.

Cohesion metrics help assess network topology by establishing whether evolutionary forces shaping metabolic relationships possess either the random, small-world or scale-free tendencies [22–24]. While the subnetwork one-mode projections did not seem to exhibit significant scale-free behavior (Table 2) or heterogeneities in the randomicity of network topology (Table 3), they displayed a significant small-world trend (Fig 8), albeit a decreasing one along the evolutionary timeline. A similar decreasing trend holds even when the age of the most ancestral domain is considered as the age of the enzyme (S9 Fig).

**Fig 9:**
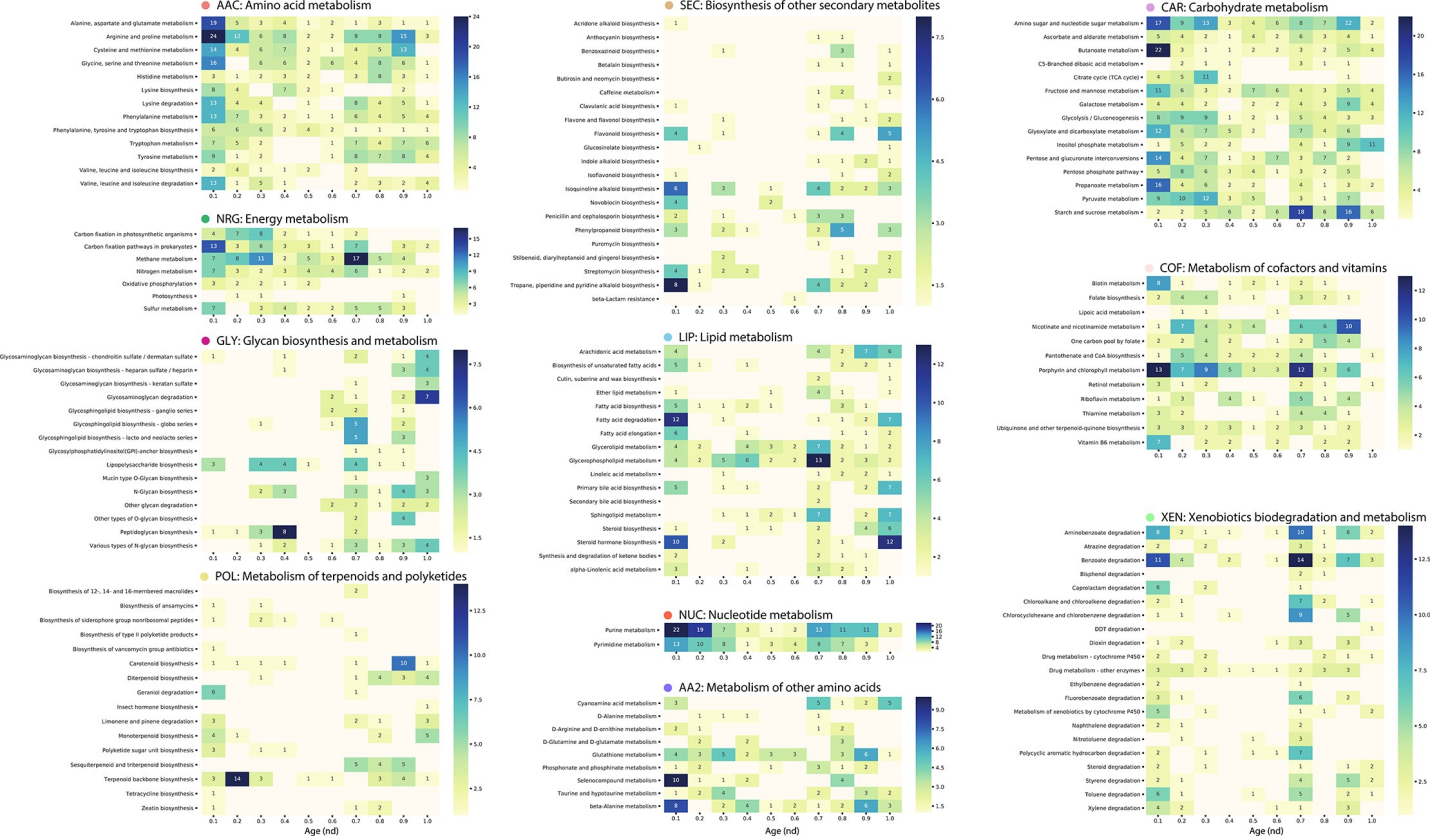
Matrix representation of subnetwork one-mode graphs by evolutionary age. Rows represent nodes (subnetworks) with each cell indicating the number of enzymes (edges) per subnetwork in each *nd* interval.

**Fig 10:**
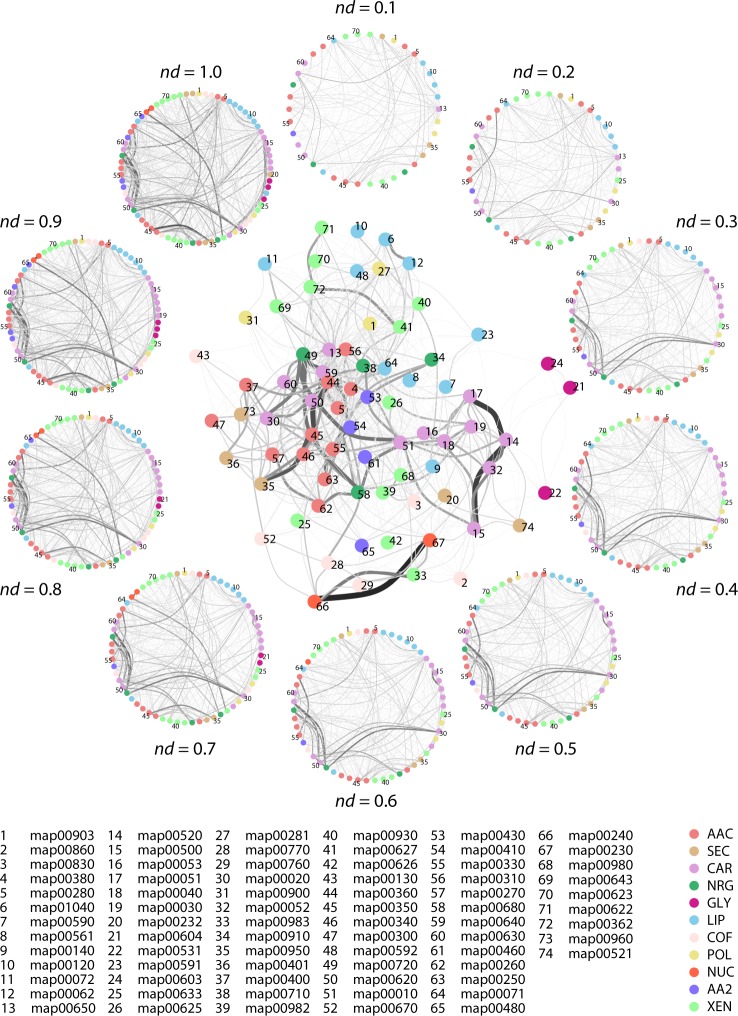
Evolution of metabolic networks visualized through the subnetwork one-mode projection of the subnetwork-enzyme bipartite network. A reduced representation of the extant subnetwork one-mode projection (*nd* = 1.0) is shown in the middle. The reduced network projection shows major nodes (subnetworks) connecting to each other through links (shared enzymes). Greyscale values of links indicate the number of enzymes shared among the subnetworks. A full description of KEGG subnetwork labels can be found in S2 Table and S3 Table. The circle of networks describes a timeline of network growth for the subnetwork projection.

Fig 9 describes the number of enzyme nodes per subnetwork, organized by mesonetwork, and plotted as function of evolutionary time with heat diagrams. The connectivity patterns of the subnetwork one-mode projection can be observed in a reduced representation (Fig 10). The nodes represent subnetworks and edges between nodes denote sharing of enzymes. The thickness of these edges as well as their greyscale color is determined by the numbers of enzymes that are shared, with black colored edges describing the highest and white colored edges the lowest levels of sharing. The reduced network representation improves the readability of the actual one-mode network (Fig 10 with labels fully explained in S2 Table and S3 Table). Significant ‘highways’ of enzyme sharing connected subnetworks of mesonetworks, supporting the hierarchical organization of KEGG. The highest connectivity levels were established between the two subnetworks of Nucleotide Metabolism (NUC), three subnetworks (map00051, map00520 and map0050) of Carbohydrate metabolism (CAR), three subnetworks of Amino acid metabolism (AAC), and two subnetworks (map00720 and map00680) of Energy metabolism (NRG). In contrast, only selected groups of subnetworks were cohesively grouped in the mesonetworks of Lipid metabolism (LIP), Metabolism of other amino acids (AA2), Metabolism of cofactors and vitamins (COF), Glycan biosynthesis and metabolism (GLY), Secondary metabolites (SEC) and Xenobiotics biodegradation and metabolism (XEN). When considering the age of an enzyme as that of its most ancestral domain, similar sharing patterns are observed (S10 and S11 Figs). The growth of the subnetwork one-mode projection reveals the gradual addition and strengthening of links between central subnetworks of the mesonetworks (Fig 10).

**Fig 11:**
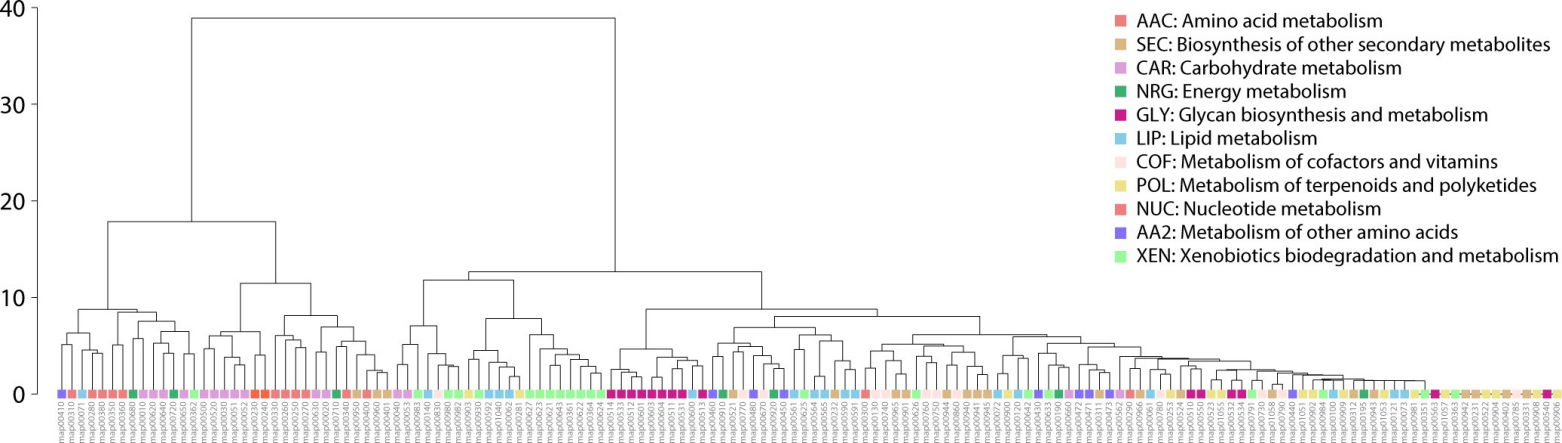
Dendrogram of the subnetwork one-mode network (at *nd* = 1.0) resulting from hierarchical clustering.

**Fig 12:**
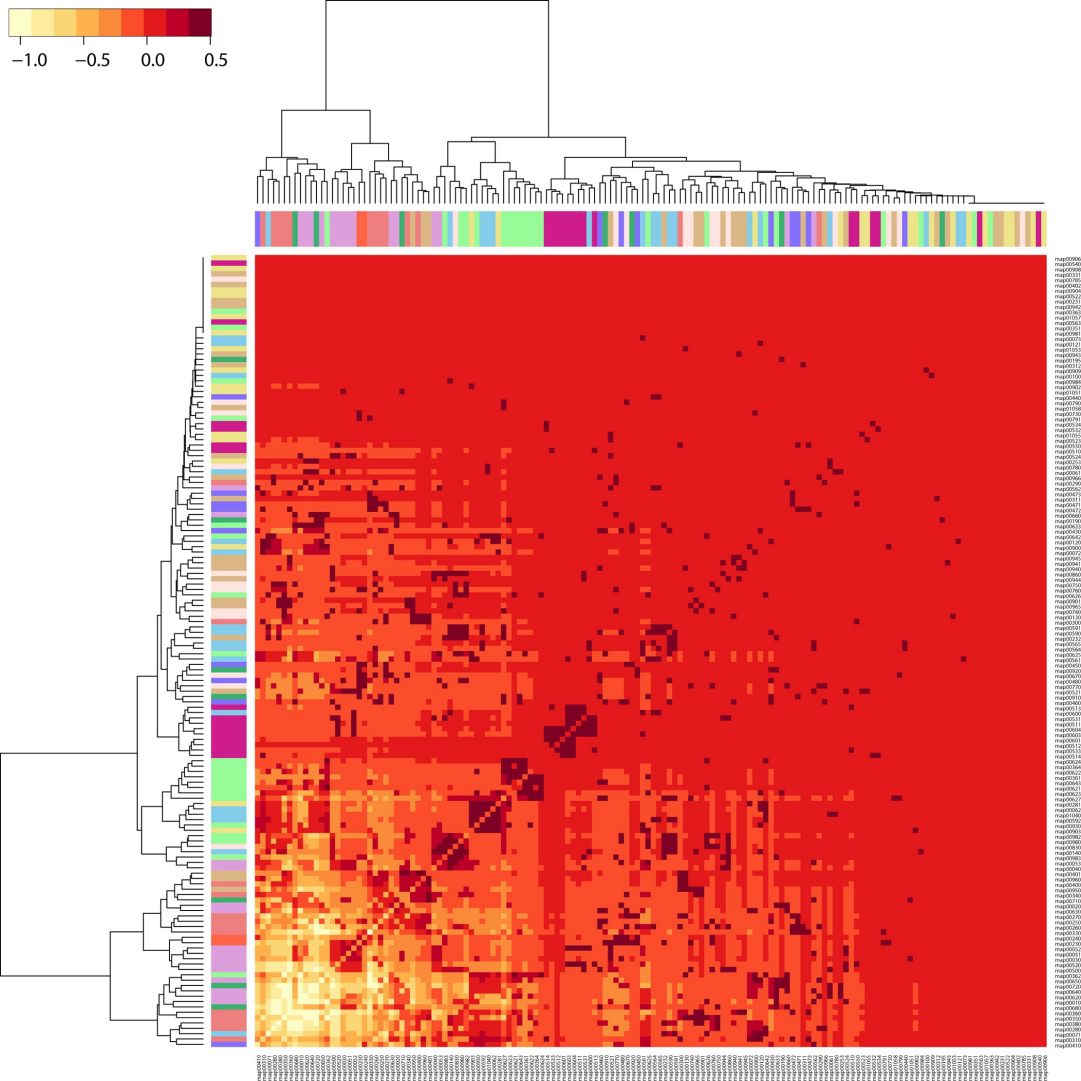
A “tapestry” of enzyme recruitment. A heatmap based on the modularity matrix was coupled to the dendrogram obtained from hierarchical clustering of the metabolic subnetworks one-mode network (shown in Fig 11).

A significant number of enzymes appear at *nd* = 0.1 populating the subnetworks associated with the amino acid (AAC), carbohydrate (CAR), energy (NRG), lipid (LIP), cofactor and vitamins (COF), and nucleotide (NUC) metabolic mesonetworks (Fig 9), supporting observations of Fig 3. The general biphasic trend of domain sharing can also be observed in most of the matrices of the mesonetworks, particularly those corresponding to secondary metabolite (SEC), glycan biosynthesis and metabolism (GLY), terpenoids and polyketides (POL), other amino acids (AA2), and xenobiotics (XEN), with high enzyme numbers in the beginning followed by a gradual decrease and then an increase at around *nd =* 0.7. The sharing of enzymes related to subnetworks for ‘Lysine biosynthesis’ and ‘Valine, leucine, and isoleucine biosynthesis’ slows down at around *nd* = 0.7, while the sharing of enzymes by their corresponding degradation subnetworks increases. The patterns of connectivity shown in the dendrogram (Fig 11) accompanied by its modularity matrix (Fig 12) indicate ‘disassortative mixing across modules’, i.e. network nodes tend to connect with other dissimilar nodes [25]. Interestingly, only the nucleotide metabolism mesonetwork (NUC) and a few subnetworks from the carbohydrate (CAR), glycan biosynthesis (GLY) and xenobiotics (XEN) mesonetworks exhibit this propensity, while the bulk of the subnetworks cluster in a heterogeneous manner.

### Enzyme one-mode projections

The enzyme one-mode networks describe subnetwork-mediated connections of enzymes. Nodes represent enzymes and edges depict enzyme sharing between subnetworks. Given the large number of nodes in this network it was difficult to visually inspect the graph for meaningful relationships. We therefore calculated cohesion and centrality metrics to uncover the existence of underlying patterns of network connectivity. To measure the propensity toward small-world properties we compared average clustering coefficients with average path lengths of the largest connected component of graphs along the timeline (Fig 8). The diameter of the network remained constant at a value of 7 throughout all the phases of the chronology (Fig 6). A small world network possesses higher clustering coefficient and a lower path length in comparison with a random network of the same size. As shown in Fig 8, the clustering coefficient steadily increased with evolutionary time with a gradual drop in average path length and a small-world coefficient that increased with time. This increasing tendency of the small-world coefficient of the enzyme projection is opposite to that of the subnetwork projection, suggesting that the two levels of metabolic organization exhibit opposing small-world trends. Like the other two networks, the behavior of the enzyme one-mode projection departs from that of the Erdős–Rényi random graph model (Table 3).

### Distribution of enzyme structures and functions among superkingdoms of life and viruses

**Fig 13:**
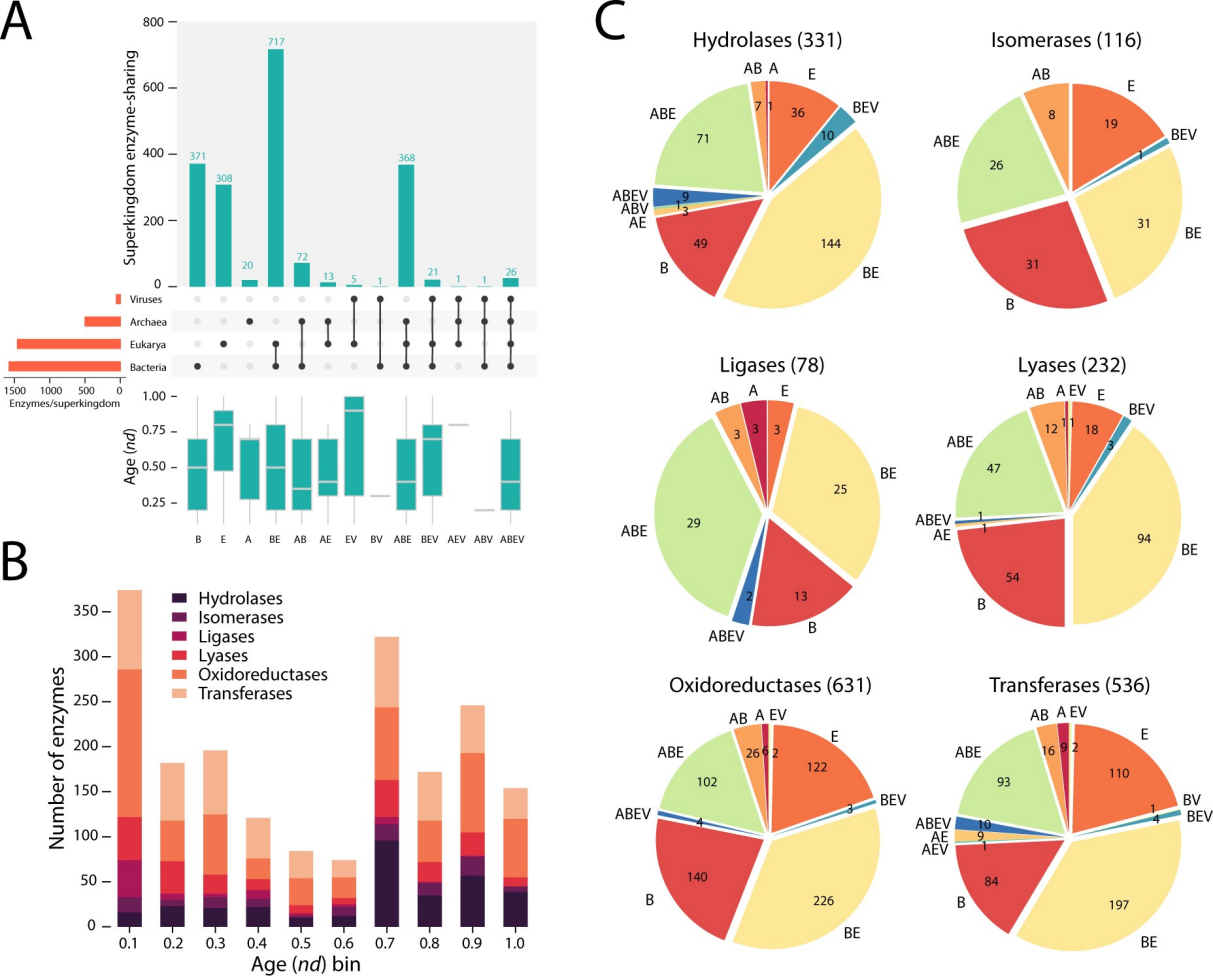
Enzyme distribution by superkingdom at EC level 1 (N = 1924 enzymes). (A) Enzyme distribution by superkingdom. (B). Enzymatic functions mapped along the evolutionary timeline. (C) EC level 1 breakdown by superkingdom. A, Archaea; B, Bacteria; E, Eukaryota; V, Viruses.

Fold superfamilies are disproportionately distributed among the three cellular superkingdoms, Archaea (A), Bacteria (B), and Eukarya (E), and viruses (V)[26]. Certain superfamilies are present exclusively in each taxonomic group (A, B, E, or V), or are found in two (AB, AE, AV, BE, BV, EV), three (ABE, ABV, AEV, BEV) or all four (ABEV) Venn taxonomic groups. The enzymes in our analysis belong to 13 out of the possible 15 Venn groups (Fig 13A). The bulk of the enzymes contain domains that are present in the BE group. The evolutionary landscape is dominated by *oxidoreductases* at *nd* = 0.1 (Fig 13B), the first peak of the biphasic “curve”, and this dominance is subsequently shared with *hydrolases* when reaching the second peak (*nd* = 0.7) of the timeline. Among the six enzyme categories at level 1 of Enzyme Classification (EC), the enzymes with domains of the BE group had the largest share in four categories, while sharing the top spot with bacterial (B) domains among *isomerases* and outnumbered by domains found belonging to the ABE group. Transferases cover most Venn groups while *lyases* were represented in the least number of them.

**Fig 14:**
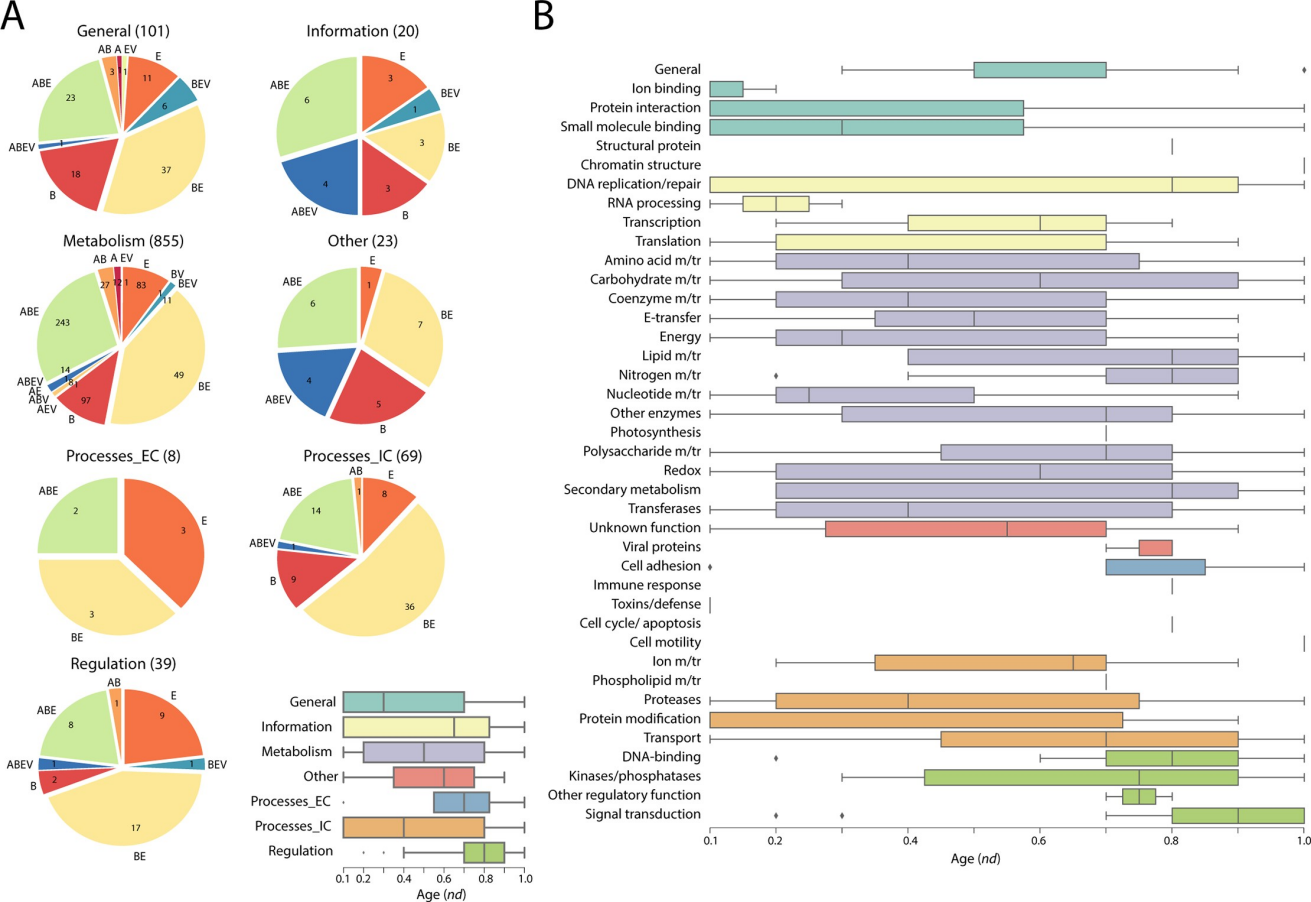
Functional distribution of enzymes. (A) Superkingdom makeup Distribution of each general functional category in superkingdoms and viruses. (B) Distribution of detailed functional categories along the evolutionary timeline.

**Fig 15:**
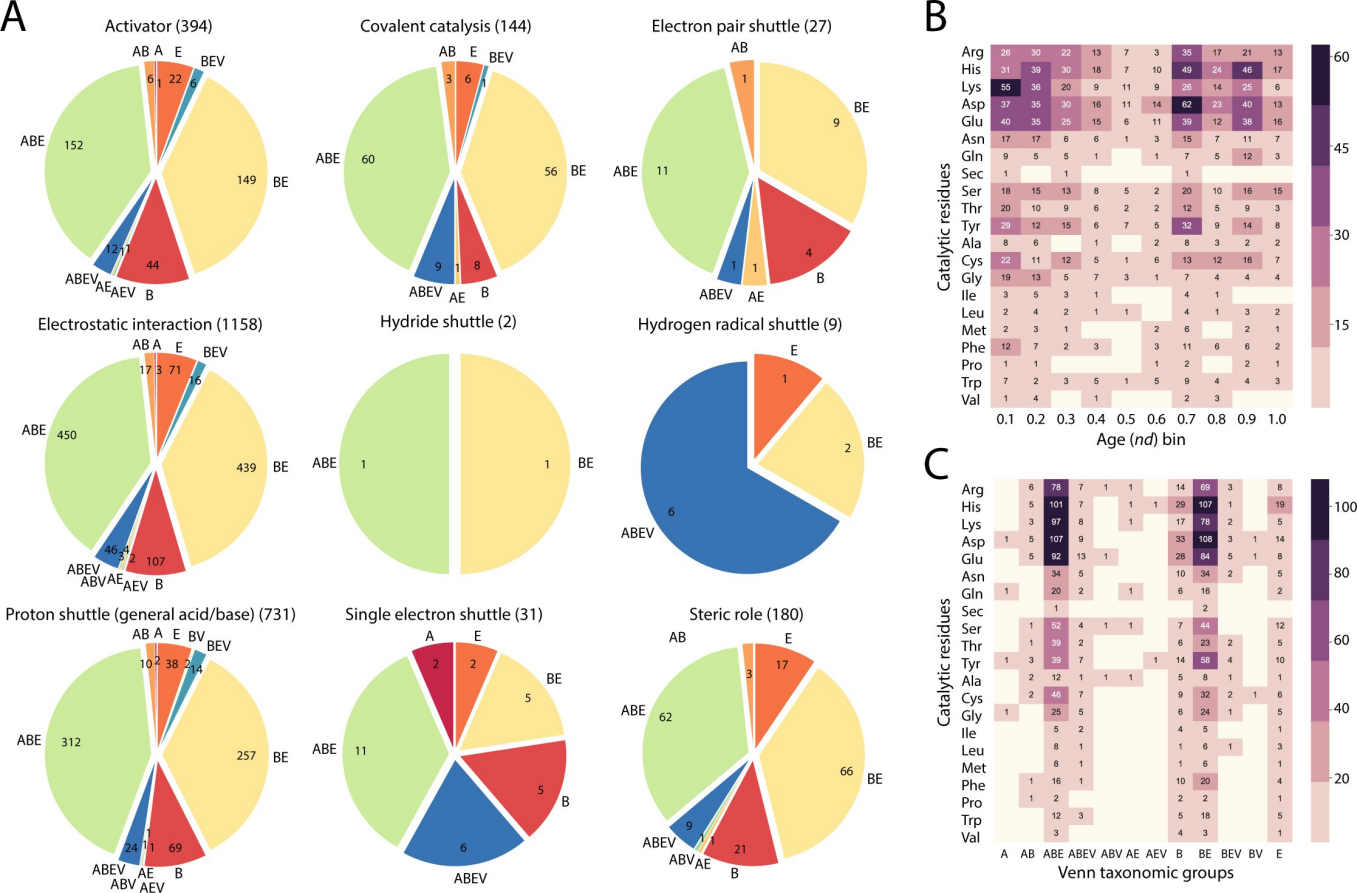
Survey of catalytic sites in all 543 enzymes of the M-CSA database that were mapped to a domain with an *nd* value. (A) Distribution of role groups of the catalytic site residues in Venn taxonomic groups of superkingdoms and viruses. (B) Distribution of catalytic residues according to when enzymes possessing these residues appeared along the evolutionary timeline. (C) Distribution of catalytic residues based on association of parent enzymes to the superkingdoms. Highlighted background indicates the group to which the amino acids belong to: purple, basic amino acids; pink, acidic amino acids; green, polar uncharged amino acids; yellow, nonpolar amino acids.

Using the functional annotation scheme of Vogel and Chothia [27], we assigned molecular functions to the superfamilies that make up the enzymes of metabolism. In this classification, molecular functions are divided into 7 ‘general’ categories, namely, *Metabolism*, *Information*, *Intracellular processes (Processes_IC)*, *Extracellular processes (Processes_EC)*, *Regulation*, *General and Other*, which are further subdivided into 50 ‘detailed’ categories. The functional annotation, which is specific to SCOP domains, has been extensively used to trace molecular functions along the timelines of domain innovation (e.g. Caetano-Anollés et al. 2011, 2012 [28,29]). As expected, the majority of the enzymes had domains that were associated with *Metabolism* (Fig 14A). The BE group dominated six general function categories in terms of number of enzymes, while having the same number of enzymes as eukaryotes (E) for Extracellular Processes. With a few exceptions, the domains annotated with *Extracellular Processes* and *Regulation* functions showed they were recruited relatively late in the timeline (Fig 14A inset). With one exception, domains belonging to *DNA-binding* of *Regulation* (Fig 14B) appeared to be recruited close to the second peak of the biphasic curve (Fig 3). The domains belonging to *Transcription* were preceded in recruitment by those belonging to *Translation* (Fig 14B). Next, we used the nomenclature put forth by Ribeiro and colleagues[30] to annotate enzymes with their reaction mechanisms and catalytic sites and study the distribution of these mechanisms and sites across the Venn taxonomic groups (Fig 15A). We found that enzymes with electrically charged amino acid residues tend to dominate the distribution of catalytic residues and do so early during the evolutionary timeline (Fig 15B), with most of them being present in domains of the ABE and BE groups (Fig 15C).

### Identifying central enzymes in metabolic networks

In order to identify some of the central nodes in the enzyme one-mode network, we calculated network metrics such as degree, closeness and betweenness centralities (Table 4). Degree centrality measures the popularity of the node with respect to how many connections it possesses. Betweenness centrality measures the “brokerage” power a node commands over the network, being present on the greatest number of shortest paths. Closeness centrality indicates how well-connected are the neighbors of a particular node, which helps in exerting power at a local level in comparison with betweenness centrality. The enzyme EC 2.3.1.9 appears to be the most well-connected and influential node in the network for degree and betweenness centralities. EC 2.3.1.9 is an acetyl-CoA C-acetyltransferase, belonging to the *transferases* class of enzymes, containing the *thiolase-related* (c.95.1.1) fold family. It catalyzes the reaction of converting two molecules of acetyl-CoA to yield acetoacetyl-CoA and CoA. Other enzymes with higher degree centralities include: aldehyde dehydrogenase (EC 1.2.1.3), aspartate transaminase (EC 2.6.1.1), alcohol dehydrogenase (EC 1.1.1.1), aldehyde reductase (EC 1.1.1.21), and enoyl-CoA hydratase (EC 4.2.1.17). The majority of the fold families of these enzymes belong to alpha and beta proteins (class c) in SCOP (Table 5).

**Table 4:** Network centrality metrics for the enzyme one-mode network extracted from the subnetwork-enzyme bipartite network.

	*nd* value										
Metric	Rank	0.1	0.2	0.3	0.4	0.5	0.6	0.7	0.8	0.9	1.0
**Degree**	1	EC 4.2.1.17	EC 2.3.1.9	EC 2.3.1.9	EC 2.3.1.9	EC 2.3.1.9	EC 2.3.1.9	EC 2.3.1.9	EC 1.2.1.3	EC 1.2.1.3	EC 1.2.1.3
	2	EC 2.3.1.9	EC 4.2.1.17	EC 4.2.1.17	EC 4.2.1.17	EC 2.6.1.1	EC 4.2.1.17	EC 4.2.1.17	EC 4.2.1.17	EC 4.2.1.17	EC 4.2.1.17
	3	EC 1.1.1.35	EC 2.6.1.1	EC 2.6.1.1	EC 2.6.1.1	EC 4.2.1.17	EC 2.6.1.1	EC 2.6.1.1	EC 2.3.1.9	EC 2.3.1.9	EC 2.3.1.9
	4	EC 2.6.1.1	EC 1.1.1.35	EC 1.1.1.37	EC 1.1.1.37	EC 1.1.1.37	EC 1.1.1.37	EC 2.7.1.69	EC 2.6.1.1	EC 5.4.2.2	EC 5.4.2.2
	5	EC 2.6.1.19	EC 1.2.7.1	EC 1.2.7.1	EC 1.2.7.1	EC 1.2.7.1	EC 2.7.1.69	EC 1.1.1.37	EC 2.7.1.69	EC 2.6.1.1	EC 2.6.1.1
	6	EC 1.1.1.1	EC 3.5.1.4	EC 1.1.1.35	EC 1.1.1.35	EC 2.7.1.69	EC 1.2.7.1	EC 2.4.1.17	EC 1.1.1.37	EC 2.7.1.69	EC 2.7.1.69
	7	EC 6.3.4.16	EC 2.6.1.21	EC 1.8.1.4	EC 1.8.1.4	EC 1.1.1.35	EC 2.4.1.17	EC 1.2.7.1	EC 2.4.1.17	EC 1.4.3.4	EC 1.4.3.4
	8	EC 1.4.1.4	EC 6.2.1.1	EC 2.4.1.17	EC 2.4.1.17	EC 2.4.1.17	EC 1.1.1.35	EC 1.1.1.35	EC 1.2.7.1	EC 1.1.1.37	EC 2.4.1.17
	9	EC 2.6.1.57	EC 2.6.1.19	EC 6.2.1.1	EC 6.3.1.2	EC 1.8.1.4	EC 1.1.1.21	EC 3.6.1.9	EC 3.6.1.9	EC 2.4.1.17	EC 1.4.3.2
	10	EC 1.1.1.157	EC 2.6.1.57	EC 4.1.2.13	EC 1.1.1.27	EC 6.3.1.2	EC 1.8.1.4	EC 2.7.2.2	EC 1.1.1.35	EC 1.4.3.2	EC 1.1.1.37
**Betweenness**	1	EC 1.1.1.21	EC 2.3.1.9	EC 2.3.1.9	EC 2.3.1.9	EC 2.3.1.9	EC 2.3.1.9	EC 2.3.1.9	EC 1.2.1.3	EC 1.2.1.3	EC 1.2.1.3
	2	EC 1.1.1.1	EC 1.1.1.1	EC 2.6.1.1	EC 2.6.1.1	EC 2.6.1.1	EC 4.2.1.17	EC 4.2.1.17	EC 4.2.1.17	EC 5.4.2.2	EC 4.2.1.17
	3	EC 2.3.1.9	EC 2.6.1.1	EC 2.4.1.17	EC 4.2.1.17	EC 4.2.1.17	EC 2.6.1.1	EC 2.4.1.17	EC 2.4.1.17	EC 4.2.1.17	EC 5.4.2.2
	4	EC 4.2.1.17	EC 4.2.1.17	EC 4.2.1.17	EC 2.4.1.17	EC 2.4.1.17	EC 2.4.1.17	EC 2.6.1.1	EC 2.6.1.1	EC 2.3.1.9	EC 2.3.1.9
	5	EC 1.1.1.35	EC 2.3.1.37	EC 1.1.1.21	EC 1.1.1.21	EC 1.1.1.21	EC 1.1.1.21	EC 3.6.1.9	EC 3.6.1.9	EC 2.4.1.17	EC 2.4.1.17
	6	EC 2.6.1.1	EC 1.1.1.21	EC 1.1.1.1	EC 1.1.1.1	EC 1.1.1.1	EC 2.3.1.37	EC 1.1.1.21	EC 2.3.1.9	EC 3.6.1.9	EC 3.2.1.31
	7	EC 2.6.1.19	EC 1.1.1.35	EC 1.1.1.35	EC 2.3.1.37	EC 2.3.1.37	EC 1.1.1.1	EC 1.1.1.1	EC 2.3.1.37	EC 2.6.1.1	EC 3.6.1.9
	8	EC 6.4.1.2	EC 6.3.5.5	EC 2.3.1.37	EC 5.3.1.1	EC 5.3.1.1	EC 2.7.1.69	EC 2.3.1.37	EC 1.1.1.1	EC 1.2.3.1	EC 3.2.1.52
	9	EC 2.3.1.37	EC 2.6.1.16	EC 5.3.1.1	EC 1.1.1.35	EC 1.1.1.35	EC 5.3.1.1	EC 1.1.1.35	EC 5.3.1.1	EC 3.2.1.31	EC 2.6.1.1
	10	EC 1.1.1.2	EC 5.3.1.1	EC 2.1.2.1	EC 1.8.1.4	EC 2.7.1.69	EC 2.6.1.16	EC 2.7.1.69	EC 1.2.3.1	EC 1.1.1.1	EC 1.1.1.1
**Closeness**	1	EC 1.1.1.1	EC 2.6.1.1	EC 2.6.1.1	EC 2.6.1.1	EC 2.6.1.1	EC 2.6.1.1	EC 1.1.1.1	EC 1.2.1.3	EC 1.2.1.3	EC 1.2.1.3
	2	EC 4.2.1.17	EC 1.1.1.1	EC 1.1.1.1	EC 1.8.1.4	EC 1.8.1.4	EC 1.8.1.4	EC 2.6.1.1	EC 2.6.1.1	EC 1.8.1.4	EC 5.4.2.2
	3	EC 1.1.1.35	EC 2.3.1.9	EC 1.8.1.4	EC 1.1.1.1	EC 1.1.1.1	EC 1.1.1.1	EC 1.8.1.4	EC 1.8.1.4	EC 5.4.2.2	EC 1.1.1.1
	4	EC 2.3.1.9	EC 2.6.1.19	EC 2.1.2.1	EC 4.2.1.17	EC 4.2.1.17	EC 1.1.1.27	EC 4.2.1.17	EC 1.1.1.1	EC 2.6.1.1	EC 1.8.1.4
	5	EC 2.6.1.19	EC 2.6.1.96	EC 2.3.1.9	EC 1.1.1.27	EC 1.1.1.27	EC 2.1.2.1	EC 2.1.2.1	EC 4.2.1.17	EC 1.1.1.1	EC 2.6.1.1
	6	EC 2.6.1.1	EC 1.2.1.24	EC 1.1.1.37	EC 1.1.1.37	EC 1.1.1.37	EC 4.2.1.17	EC 1.1.1.21	EC 2.1.2.1	EC 1.4.3.4	EC 1.4.3.4
	7	EC 2.6.1.96	EC 4.2.1.17	EC 2.6.1.19	EC 2.1.2.1	EC 2.1.2.1	EC 1.1.1.37	EC 1.1.1.27	EC 5.3.1.9	EC 2.7.1.40	EC 2.7.1.40
	8	EC 1.2.1.24	EC 2.1.2.1	EC 1.1.1.27	EC 2.3.1.9	EC 2.3.1.9	EC 1.1.1.21	EC 2.7.1.31	EC 2.7.1.31	EC 1.1.1.27	EC 2.3.1.9
	9	EC 6.2.1.1	EC 6.2.1.1	EC 2.6.1.96	EC 2.6.1.19	EC 1.1.1.21	EC 2.3.1.9	EC 2.3.1.9	EC 2.7.1.165	EC 2.3.1.9	EC 2.7.1.1
	10	EC 2.1.2.1	EC 1.2.7.1	EC 1.2.1.24	EC 1.1.1.21	EC 2.7.1.31	EC 1.2.1.16	EC 1.1.1.37	EC 1.4.3.21	EC 1.4.3.2	EC 1.4.3.2

**Table 5:** Enzymes with high network centrality metrics in the enzyme one-mode network that were extracted and their corresponding SCOP concise classification strings (ccs).

EC Number	SCOP ccs	SCOP Fold Family Description	*nd* value
EC 1.1.1.1	b.35.1.2	Alcohol dehydrogenase-like, N-terminal domain	0.0985401
	c.2.1.1	Alcohol dehydrogenase-like, C-terminal domain	0.0985401
	c.2.1.2	Tyrosine-dependent oxidoreductases	0.00364964
EC 1.1.1.21	c.1.7.1	Aldo-keto reductases (NADP) domain	0.0985401
EC 1.2.1.3	a.23.4.1	Mitochondrial import receptor subunit Tom20	0.781022
	c.82.1.1	ALDH-like domain	0.0291971
EC 2.3.1.9	c.95.1.1	Thiolase-related domain	0.0912409
EC 2.6.1.1	c.67.1.1	AAT-like domain	0.0364963
	c.67.1.3	Cystathionine synthase-like domain	0.0364963
EC 4.2.1.17	a.100.1.3	HCDH C-domain-like domain	0.791971
	c.14.1.3	Crotonase-like domain	0.0912409
	c.2.1.6	6-phosphogluconate dehydrogenase-like, N-terminal domain	0.080292
	c.95.1.1	Thiolase-related domain	0.0912409
	d.38.1.4	MaoC-like domain	0.813869

## Discussion

### MANET 3.0 dissects metabolic history at fold family level

The new release of metabolic MANET significantly expands the number of indexed enzymatic entries (Table 1). This can be attributed to increases in SCOP and KEGG records, links between enzyme and PDB information provided by inclusion of a fourth data source, PDBsum indexing, and improvements in hidden Markov model (HMMs) methodologies for predicting protein domain structures. More importantly, MANET 3.0 now traces evolution of structural domains at fold family level enhancing the evolutionary tracings of previous versions, which originally focused on structure defined at fold level of SCOP classification. In comparison with folds, SCOP families are highly conserved at sequence level and represent cohesive units of functional similarity. They describe molecular functions in more detail than folds and their evolutionary relatedness can better dissect evolutionary recruitment. For example, the use of fold families improved the evaluation of domain gain and loss in proteomes, revealing the primacy of domain gain in evolution and pervasive tendencies of proteomic growth [31]. Therefore, fold families are better equipped for studying evolution of proteins and corresponding molecular functions, which are deeply embedded in protein structure [32].

The timelines of structural domains and the validity of age assignments have been tested in a number of studies. Known transformation pathways describing evolutionary changes in fold structure [33] support the domain timeline [2]. Examples include the gradual effect of insertions/deletions (indels) and substitutions in the Rossmann-like fold structure of synapsin or the transformation of an all-α 3-helical bundle into an all-β barrel-like structure involving the C-terminal CAP domain. The age of domains has been shown to be proportional to geological time for SCOP folds and fold superfamilies [34] and for fold families [5,29], provided fold families were the most ancient in each fold superfamily. The strong correlations define a universal molecular clock of protein structure capable of tracing the early history of aerobic metabolism and planet oxygenation [34] or the early and concurrent origin of cysteine biosynthesis and iron-sulfur proteins [35] Similarly, a strong correlation between the history of domains and the history of RNA structural components of the ribosome mutually validated age assignments, supporting the ancient co-evolution of ribosomal proteins and RNA [36]. Furthermore, the elaboration of an evolutionary model of early biochemistry that is firmly grounded in phylogenomic information and biochemical, biophysical, and structural knowledge provided additional validation support [29]. Finally, modeling and simulations made explicit the link of phylogenomic tree imbalance and protein structural innovation, which suggests that exploration of the space of protein structure occurs through coarse-grained discoveries that undergo fine-grained elaboration [37].

### Evolutionary patterns of enzyme recruitment in metabolic networks

An initial coarse-grained analysis of MANET 3.0 provided significant insights into the origins and evolution of metabolic networks. The analysis assigned evolutionary age to a multidomain enzyme based on the second most ancient domain it contains. However, age assignments based on the most ancient domain of the enzyme [16] (which can be found in S4–S13 Figs and S4–S6 Tables) did not change evolutionary trends and conclusions. These age assignments assume that proteins evolve by mutation, duplication, amplification, recombination, and *de novo* creation of genes and that accretion of domains in multidomain proteins occurs fundamentally by fusions of domains to already functional structural units [31]. Both assumptions are well supported, especially because a careful mechanistic study of domain fusions and fissions in the proteins of hundreds of proteomes showed that fusions override fissions throughout the timeline and that fissions occurred relatively late in protein evolution [38].

On a global level, the burst of enzymatic innovation observed in our analysis of enzyme distributions along the evolutionary timeline (Fig 4) matches previous reports suggesting the existence of a ‘big bang’ of functional innovation in metabolism [4,39]. In this regard, we find that “ancestral” enzymes (*nd* = 0.1, S5 Fig) are prevalent when these are defined by the age of the oldest domains and despite the decline of this initial burst with evolutionary time (S5 Fig). We also find there was no clear sequential pattern of evolution of enzymes in metabolic pathways of the bipartite networks that describe the sharing of enzymes between mesonetworks and subnetworks. Instead, pathways in metabolic subnetworks revealed a ‘patchwork’ of enzymes, i.e. a heterogeneous ensemble of enzymes of different age (Fig 11 and Fig 12). These observations provide strong support to the patchwork model of metabolic evolution. Our observation of high levels of sharing of enzymes in the carbohydrate and amino acid metabolism mesonetworks is supported by earlier studies in which *nd* values of domains defined at fold superfamily level were used [6,39].

We find that the majority of the domains recruited by the enzymes of nucleotide metabolic pathways were ancestral, appearing early in the timeline (*nd* = 0.1 and *nd* = 0.2). This is supportive of other studies that suggest enzymatic metabolism originated in the pathways of nucleotide interconversion of the purine metabolism subnetwork [5] as part of the repertoire of ancient mesonetworks of metabolism [4]. This early seeding of enzymes of the nucleotide metabolism mesonetwork was followed by gradual build-up of pathways of biosynthesis, catabolism and salvage of nucleotides. This is also supported by the majority of domains involved in *RNA processing* and *Transcription* recruiting other domains later along the timeline (Fig 14B). These results suggest a protein world of emergent structural domains preceding an RNA world in an origin of life scenario informed by phylogenomics that has considerable explanatory power [5]. Another notable result is the significant number of enzymes arising from the lipid metabolism mesonetwork (*nd* = 0.1), in comparison with the entire cohort of enzymes (Fig 3). These evolutionary tracings suggest the centrality of the cellular makeup during the early evolutionary stages of metabolism development, which is in line with the concept of metabolic ‘shells’ put forth by Morowitz [40]. According to this hypothesis, the most ancient metabolic shell consists of a catalytic “energy amphiphile” core encompassing chemical reactions from the glycolysis, citric acid cycle, and fatty acid biosynthesis subnetworks. This initial core was followed by the inception of a second layer comprising metabolic pathways of amino acid synthesis. This follows Hartman’s speculation that the citric acid cycle appeared earlier than amino acid metabolism during evolution, which preceded the development of nucleotide biosynthesis [41]. Morowitz’s third and fourth shells endowed metabolism with the capabilities for transfer of sulfur to sulfur-containing amino acids (cysteine and methionine) and ring formation processes required for the biosynthesis of purine and pyrimidine bases. The early accumulation of enzymes in the carbohydrate, amino acid and lipid mesonetworks (Fig 3) and the late development of biosynthetic pathways of nucleotide metabolism [5] are in accordance with the metabolic shell hypothesis. The hypothesis also draws support from the analysis of the metabolic subnetwork wheel for the P-loop hydrolase fold, which is suggestive of the citric acid cycle being ancestral among all the metabolic subnetworks [4]. This pattern is evident in the grouping of subnetworks in the metabolic subnetwork wheel for the TIM β/α-barrel fold [39]. It is remarkable how the modern evolutionary patterns of metabolism are reflective of those from the prebiotic world, especially when considering that Morowitz’s concept of shells was put forth to explain a prebiotic world without enzymes. Thus, modern metabolism should be considered a “palimpsest” of ancient (perhaps global) prebiotic metabolisms.

The pyrimidine metabolism subnetwork appears to have recruited enzymes from the more primordial purine metabolic pathway [5]. This early pattern of recruitment of the nucleotide metabolism mesonetwork manifests in significant sharing of enzymes between these two subnetworks when compared to any two other subnetworks (Figs 9 and 10). This initial evolutionary patchwork of sharing that appears for example in patterns of hierarchical clustering (Fig 11), entails enzyme recruitment occurring in very early metabolic pathways of modern metabolism (Fig 12). These early patterns later on manifested into a dense “tapestry” of enzymatic sharing mediated by recruitment. The distribution of enzymes, as seen in the clustering results, also underpins the conclusions drawn from our network visualizations. The noticeable exchange of enzymes among carbohydrate, amino acid and energy metabolic pathways forms a separate cluster (Fig 11). This cluster is largely composed of ‘core enzymes’, shared largely between amino acid metabolism (AAC) and carbohydrate metabolism (CAR). These enzymes have been previously found to be conserved and produce a wide range of substrates [42]. Unlike ‘core enzymes’, enzymes and pathways related to Xenobiotics biodegradation and metabolism (XEN) and Glycan biosynthesis and metabolism (GLY) clustered at the periphery of the network (illustrated in S14 Fig). In contrast, the nucleotide metabolism mesonetwork (NUC) clustered within the core. Peripheral pathways and enzymes reflect innovation at the organismal level and do not possess flexibility in production of substrates [42]. Interestingly, clustering of xenobiotic degradation pathways into a cohesive group transcends mesonetwork boundaries by sharing with other pathways at a relatively higher level in the hierarchy. It also indicates how clusters may be acting in concert for specific functions [43]. Additionally, the hierarchical clustering patterns we observe define a modular hierarchical community structure with foundations in functionality [21].

Intriguingly, 7 out of the 21 subnetworks from the secondary metabolites mesonetwork (SEC) covered in MANET 3.0 appear relatively recently in evolution. Secondary metabolism is believed to have originated from primary metabolism to equip organisms with a “selective advantage” for survival, such as providing capabilities for antibiotic resistance in bacteria or chemical idiosyncrasies in plants [44]. These adaptation-driven pathways, which become active upon the availability of requisite substrates [45], explain the lack of connectivity of subnetworks developing late in evolution. The absence or limited sharing of enzymes with other subnetworks likely stems from biochemical specialization as the modern unfolding pathways confer individual beneficial properties to specific groups of organisms.

Finally, enzymatic, functional and catalytic site distributions among superkingdoms (Figs 13A and 14A) suggest that the common ancestor of life had a complex repertoire of metabolic domains and a complete set of functions [46][4]. The ancient innovation in enzymatic functions show the biphasic pattern of diversification at the beginning of the timeline, followed by a decrease in diversity with time (Fig 13B and Fig 14)[17].

### Evolution of metabolic network structure

MANET embeds enzymes into subnetworks and subnetworks into mesonetworks according to the knowledge-based classification scheme of KEGG. To study the evolutionary emergence of this hierarchical structure, we traced the evolutionary growth of bipartite networks and their one-mode projections. Bipartite networks are uniquely fit to study the evolutionary structuring of metabolism. They can provide remarkable insights into network connectivity. For example, the enzyme projection of the bipartite network of enzymes and subnetworks shows how subnetworks are capable of structuring the emerging world of metabolic enzymes. In turn, the subnetwork projection reveals how the world of subnetworks is structured by the sharing of enzymes. In other words, network projections make explicit how hierarchy and modularity unfold at each level of metabolic organization (Fig 2).

The evolving structure of our metabolic networks revealed hierarchy and modularity unfolding at each organization level. Patterns of enzyme sharing along the evolutionary timeline displayed a tendency toward power law in the bipartite networks (Table 2). Such a tendency has been previously described [20,47]. However, both subnetwork and enzyme one-mode network projections showed small world-like behavior. This behavior is congruent with metabolic studies that highlight the small world properties of the metabolite and reaction relationship [48]. Many biological networks exhibit high clustering coefficients, an observation that suggests their modularity is hierarchically structured [49]. As expected, the mean clustering coefficients for subnetwork and enzyme one-mode projections ranged 0.5–0.6 (Fig 8A), in congruence with previous analyses of metabolic networks [21]. While hierarchical modularity has been observed in scale-free networks and does not entail a cause-and-effect relationship between them [50], the gradual rise of the clustering coefficient in the enzyme one-mode networks provides strong support to the gradual evolutionary development of hierarchical modularity in metabolic networks. Strikingly, the clustering coefficients of the enzyme one-mode network projections scaled with connectivity according to a power law (Fig 7), which constitutes a hallmark of the hierarchical modularity property of complex systems (e.g. metabolism; [21,51]). This power law scaling relationship strengthened with time. In contrast, the scaling relationship of the subnetwork one-mode projections was weaker, suggesting evolutionary constraints loosen at higher metabolic hierarchy levels. Thus, hierarchy and modularity emerge more strongly at lower levels of network organization, while at the same time providing the higher organization levels with ample room for metabolic innovation.

Hierarchical modularity has been shown to increase with time in evolving biological networks at different timescales, from nanosecond-scale dynamics of the folding of loop regions in proteins to a scale of billions of years of history protein structure and function [52]. The rise of hierarchical modularity embodies a biphasic model that explains the origin and evolution of modules [17]. At first, parts (nodes) of a network describing a system such as metabolism are weakly linked. This lack of interaction between parts enables their diversification when parts are subjected to mutation, recruitment and reassortment. Diversification results in parts competing with each other through *competitive optimization*, which leads to a decrease in overall diversity and a hierarchical structuring of emerging modules. The modules that arise from the optimization process are resilient to change and increase linkage, contributing higher and higher levels of structure to the evolving system. MANET data is compatible with a similar biphasic model of diversification and unification. The model is at work in metabolism in the form of: (i) steadily low-high fluctuating diameter and high modularity scores in the enzyme and subnetwork projections (Fig 6), and (ii) the rise of a strong scaling signal of hierarchical structure operating at the lowest enzyme level of metabolic organization (Fig 7). The subnetwork projections are the source of noise in the metabolic system while the enzyme projections constrain these effects at the evolutionary level. This antagonistic effect on structure confers both flexibility and robustness to the evolving bipartite network. ‘Messiness’ is a source of novelty in evolution [53]. While evolution does not explicitly foster messiness, it exploits it for biological innovation. In metabolism, the noisy patterns we observe in the subnetwork projections suggest that noise acts as a source of innovation in metabolism that mirrors the buffering effect of the enzyme projection on the emergent subnetwork-enzyme structure of the bipartite network.

### Metabolic centralities

We found that transferase activity EC 2.3.1.9 was the most prevalent enzyme function based on degree and betweenness centrality measures of the enzyme one-mode network projections. The enzymes with this enzymatic function comprise the thiolase-related fold family (c.95.1.1; nd = 0.091). These acetoacetyl-CoA C-acetyltransferase enzymes participate in a reaction that catalyzes the formation of acetoacetyl-CoA from two molecules of acetyl-CoA, which is central for lipid metabolism. Other enzymes of significant centrality include aspartate transaminase (EC 2.6.1.1), which holds the cystathionine synthase-like family (c.67.1.3) and AAT-like family (c.67.1.1) domains, both of an age of nd = 0.036. The enzyme catalyzes reactions that result in the production of the hub metabolite glutamate. Similarly, an oxidoreductase yielding the hub metabolite NADH as one of the reaction products (EC 1.1.1.1), with its tyrosine-dependent oxidoreductase family domain (c.2.1.2, nd = 0.004), was also among the top nodes with high centralities. In the most ancient enzyme one-mode projections (S7 Table), centrality measures revealed that oxidoreductase activity EC 1.2.1.3 was the most prevalent enzymatic function, while also being one of the top nodes based on the second most ancestral age criterion. The enzymes with this enzymatic function comprise two domains, one being the aldehyde reductase fold family (c.82.1.1; nd = 0.029). The c.82.1.1 family is found in enzymes responsible for energy interconversion pathways of purine metabolism and is the most ancestral among the domains of this subnetwork, which is the oldest of metabolism [5]. One of the reactions it catalyzes yields as one of its by-products the hub metabolite NADH. Hub metabolites may be key to evolution of the most recent metabolic pathways by promoting recruitment of enzymes [54]. Along with evidence of patchy recruitment of metabolic enzymes throughout evolution, our findings also account for the high connectivity of these enzymes in our network. The *nd* values of the central enzymes, majorly EC 2.3.1.9, suggest that CoA acting as a hub metabolite is more ancestral in terms of recruitment ages. Additionally, other findings from our enzyme one-mode networks corroborate results from a previous study that show common metabolic substrates are highly connected in the networks [20]. Nonetheless, it may be significant to investigate enzymes with higher betweenness centrality that may have relatively lesser connections with other enzymes within the same network but more across the networks and modules, since such enzymes have been found to be evolutionarily conserved [43].

### The challenges of metabolic retrodiction

The deep molecular exploration of the past is challenging. Its accuracy rests on the validity of structural and functional knowledge. The accuracy of the metabolic tracings of MANET 3.0 depends on the accuracy of the SCOP, KEGG and PDBsum databases that form the bulk of its foundational elements, including biases in sampling and the definitions of fold families, enzyme activities, subnetworks and mesonetworks [2]. These possible limitations may be illustrated by the observation that folds, often treated as discrete units, may exist in a continuum in sequence space [55]. In this regard, MANET relies on the monophyletic nature of domains defined at some higher abstraction level of SCOP, i.e. the structural classification and structural variant categorization that place domains into discrete structural and evolutionary units [55]. This can be affected by the “continuous” nature of some regions of protein sequence space. Similarly, artifacts and experimental biases introduced by crystallography and molecular biology operations can also be important. The majority of the protein enzymatic entries addressed by metabolic MANET are globular in nature. Therefore, membrane proteins and other proteins that pose difficulty in experimental resolution of structures will likely be underrepresented in the databases and HMM libraries. Finally, HMM predictions are robust despite not being based on experimental data due to their satisfactory performance in comparison with structural methods [6]. Finally, caution needs to be exercised when interpreting results from phylogenomic methods. While phylogenomics is a powerful tool in the evolutionary bioinformatics arsenal [8], phylogenetic features (characters) that are studied are structural properties of present-day molecules [5]. These characters are not molecular features that existed in the distant past, but rather a representation of living molecular fossils. Despite all of these possible limitations, biases may not drastically affect the historical signal that is present in phylogenomic data [3]. Instead, increases in knowledge and database depositions with time will improve the accuracy of data, findings and conclusions.

MANET 3.0 supplements existing data-mining and other exploratory strategies that address questions and theories supporting the study of both the origin and evolution of modern metabolism and life. Potentially, MANET could be used to solve intricate questions such as the centrality of the reductive (reverse) citric acid cycle (rTCA) at the heart of the prebiotic metabolism leading to primordial amino acid and nucleotide biosynthesis pathways [56], the origin of metabolic pathways responsible for photosynthesis [57], the role of ATP synthesis behind the existence of chloroplast and mitochondrial genomes [58], the rise of planetary oxygen on Earth [59], or the presence of modularity in metabolism as design of function [43]. The classification of substrates and their relationship to metabolites of enzymes [21] could be investigated from an evolutionary perspective. Additionally, there may be merit in studying evolutionary patterns of metabolism at the fold superfamily level and how these relate to those of folds (MANET 1.0 and 2.0) and fold families (MANET 3.0). The degree of conservation offered at the superfamily level may provide the right resolution to dissect other important drivers of metabolic evolution. Investigating these evolutionary drivers in organisms representative of the three superkingdoms [20,43] could indicate whether such processes are ubiquitous across the major superkingdoms of life. Lastly, extending the metabolic MANET to cover signaling networks could enhance our understanding of evolutionary patterns in biological communication.

### A principle of increasing granularity in hierarchical structure

We have dissected the interface of different levels of hierarchical organization that are typical of complex molecular systems. The main take home message of our study of metabolic evolution is the likely existence of a *‘principle of granularity’*, an increase of the cohesiveness of lower-level parts of a system. The evolutionary trends in modularity (Fig 6), power law scaling of network clustering (Fig 7), and small-world coefficient (Fig 8) confirm Herbert A. Simon’s prediction: *“Each of the parts of a nearly-decomposable system has strong internal links among its sub-parts*, *but the several top-level parts are bound together with each other only by comparatively weak linkages”*[60]. Indeed, the evolutionary constraints on metabolic structure are stronger at the ‘enzyme’ lower level and weaker at the ‘subnetwork’ higher level of metabolic organization. The lower level structure is maintained tightly knit by a set of enzymes with significant centralities in the metabolic network (Tables 4 and 5). Such *‘tela vitae’* increases the granularity of the metabolic system by fostering interactions mediated by enzymatic recruitments that are evolutionarily entrenched by catalytic functionalities. In contrast, upper levels of network structure are bound together by linkages that are weak and more chaotic, as exemplified by our tests of network randomness (Table 3). This looseness in the establishment of pathways of chemical reactions manifests in significant heterogeneities of enzyme sharing across superkingdoms and viruses (Fig 13), diverse mapping of enzymatic functionalities (Fig 14), and cooption of catalytic site diversity (Fig 15) along the evolutionary timeline.

Simon [61] was inspired by theoretical analysis of economic, physical and biological systems. His prediction about the ‘parts-within-parts’ structure of systems justified the widely adopted assumption that most systems are near-decomposable, i.e. they have parts that mostly act independently from each other. Our principle of granularity now explains near-decomposability as the result of a trend of the system’s lower levels to become increasingly more granularly entrenched with time. We find that high granularity levels endow systems with an architecture of parts acting almost independently from each other.

## Methods

The data for the metabolic MANET 3.0 update comprises four sources. The first source, KEGG (69 version), is a compendium of metabolic network information [10]. It was used to borrow pathway and enzyme-related information. SCOP, the second source, includes a hierarchical classification of protein domains [62]. The latest SCOP release (1.75 version) provided PDB information linked to the corresponding fold families used in MANET. Kim *et al*. [63] reconstructed a phylogenomic timeline of fold families based on 989 proteomes, which was used to determine domain age of the respective enzymes containing these structural units of proteins. The underlying assumption of this phylogenomic timeline is that the most ancient domains are the ones that are most abundant and spread in nature (found in most of the proteomes). The age of the domains is expressed in terms of a ‘node distance’ (nd) value on a continuous scale of 0 to 1 that establishes the relative age of individual domains, from ancient to recent. Finally, the PDBsum repository of 3D structural information of metabolic enzymes [14], was used in addition to the three sources used in the previous version of MANET. The Enzyme Structure Database, which is part of PDBsum (release September 21, 2013), provided PDB information linked to enzymes. This information is not supplied anymore by the relevant enzyme flat file of the KEGG database.

The massive amount of data obtained from these sources was parsed to extract relevant information with Python scripts. Database management was implemented with MySQL. Join operations in MySQL helped integrate the core of MANET comprising of pathways, enzymes, PDB structure entries and fold family classification (Fig 1). A separate relation containing amino acid sequences of the enzymes was derived from the KEGG Genes data file. These amino acid sequences were used as input for generating fold family or PDB assignments via Hidden Markov Models (HMMs) of enzymes that lacked this information in the KEGG data files. The SUPERFAMILY database was scanned using HMMER, using an e-value of 0.0001 [64,65]. This annotated information facilitated an increased enzyme coverage with a median painting efficiency of 77.53% [6], which was added to the core database relation of MANET. Finally, the ancestry of the enzymes was literally painted onto the KEGG metabolic maps for visualization of the data contained in MANET, using the Python Imaging Library (PIL) [66]. The spectrum of colors, representative of ancestry, complied with the coloring scheme in the previous database version, with red to blue indicating ancient to recent, respectively. The interface design of the website in PHP of the previous version was retained, rendering the maps as interactive entities.

Network analysis of this dataset was performed by constructing bipartite networks at the mesonetwork and metabolic subnetwork level. The respective bipartite networks showcased the global patterns of connectivity existing in our data. The network construction, analysis of metrics of connectivity, and hierarchical clustering was performed using ‘igraph’ in R[18]. The power law fit was evaluated using the respective function in ‘igraph’ using the ‘plfit’ implementation that applies the Kolmogorov-Smirnov test to the data. The Bartels’ ranked test for randomness was performed using the R package ‘randtests’ using ‘two.sided’ as the alternative hypothesis and ‘beta’ p-values [67]. To assess small-world behavior, we calculated the small-world coefficient, *S*^Δ^, by comparing it with an equivalent Erdös-Rényi random graph generated using igraph’s ‘erdos.renyi.game’ function. Using igraph’s inbuilt functions to calculate the average clustering coefficient, $C_{g}^{\Delta }$, of the graph (bipartite network or either of the one-mode projections) and average path length, *L_g_*, as well as the average clustering coefficient, $C_{rand}^{\Delta }$, and average path length, *L_rand_*, of an equivalent Erdös-Rényi random graph [68] we calculated the small-world coefficient, *S*^Δ^, by:

$$S^{\Delta }=\frac{\gamma_{g}^{\Delta }}{\lambda_{g}}$$

where

$$\gamma_{g}^{\Delta }=\frac{C_{g}^{\Delta }}{C_{rand}^{\Delta }}$$

$$\lambda_{g}=\frac{L_{g}}{L_{rand}}$$

The patterns of connectivity shown in dendrograms (Fig 11) and associated modularity matrices (Fig 12) were created by computing dissimilarities via squared Euclidean distances. They were hierarchically clustered with Ward’s algorithm [69]. The heatmap was generated using a modularity matrix calculated by the Fast Greedy Community method and scaled to range between -1 and 1.

Superkingdom and viral annotations of enzymes were obtained by mapping EC numbers to species data from BRENDA release 2018.2 (available at www.brenda-enzymes.org) [70], which were traced onto superkingdom data from the NCBI taxonomy database [71]. In order to identify catalytic sites, we mapped EC numbers to all catalytic sites of the 543 enzymes covered in the M-CSA that was mapped to a domain with an *nd* value [30]. Functional annotations of enzymes were derived by mapping SCOP FSF IDs onto SUPERFAMILY functional categories [27]. Note that EC numbers have been revised after the creation of MANET. Annotations associated with the revised names were used while retaining the old names to maintain correspondence with maps on MANET (and data associated with the KEGG 2014 release) (S4 Table). In the case of an EC number being transferred to multiple revised EC numbers, the superkingdom annotations were only used if there were an absolute consensus among the annotations of all the revised EC numbers. This resulted in EC 1.7.99.4 being excluded from superkingdom annotation analysis.

## Supporting information

S1 Fig Painting efficiency of the subnetworks from MANET and KEGG. The x-axis represents the subnetwork number, while the y-axis (left) denotes number of enzymes per subnetwork. The y-axis (right) indicates percentage trend of the coverage.
(TIF)

S2 Fig Log-log plots for the bipartite network derived from subnetwork-enzyme connections at different *nd* values (*nd* age of multidomain enzymes using the age of the 2nd most ancient domain). (TIF)

S3 Fig Number of nodes in each network type at 0.1 *nd* intervals (*nd* age of multidomain enzymes using the age of the 2nd most ancient domain). (TIF)

S4 Fig A bipartite graph of mesonetworks and enzymes that are extant (nd = 1.0) showing enzymes by nd distribution (*nd* age of enzymes using the age of the most ancient domain), on a scale of red to violet representing ancestral to recent fold family domains assignments respectively. Mesonetwork are shown as open circles while colored nodes denote enzymes.
(TIF)

S5 Fig Run chart of enzymes in mesonetworks appearing in each *nd* era (*nd* age of enzymes using the age of the most ancient domain). Eras are defined as *nd* bins of ages; the first *nd* bin includes enzymes appearing between *nd* = 0 and *nd* = 0.1. The inset describes the distribution of enzymes along the evolutionary timeline.
(TIF)

S6 Fig Connectivity patterns among mesonetworks at different stages of the evolutionary timeline (*nd* age of enzymes using the age of the most ancient domain). Mesonetworks are represented by vertices while edge thickness shows the number of enzymes shared. AAC, Amino acid metabolism; SEC, Biosynthesis of other secondary metabolites; CAR, Carbohydrate metabolism; NRG, Energy metabolism; GLY, Glycan biosynthesis and metabolism; LIP, Lipid metabolism; COF, Metabolism of cofactors and vitamins; POL, Metabolism of terpenoids and polyketides; NUC, Nucleotide metabolism; AA2, Metabolism of other amino acids; XEN, Xenobiotics biodegradation and metabolism.
(TIF)

S7 Fig Average node degrees (average number of links), diameter and maximum modularity scores for each type of network (largest connected component) at each time point (0.1 *nd* interval; *nd* age of enzymes using the age of the most ancient domain). Network sizes (total number of nodes and nodes in the largest connected component) are given in S12 Fig.
(TIF)

S8 Fig Log-log plot of *C(k) vs k* for the one-mode enzyme projections at *nd* value intervals of 0.1 (*nd* age of enzymes using the age of the most ancient domain). (TIF)

S9 Fig Testing for small-world behavior in Enzyme and Subnetwork one-mode networks. (A) Comparison of clustering coefficient and average path length of the enzyme one-mode network to that of an Erdős–Rényi network. The resulting small-world coefficient seems to increase along the evolutionary timeline (*nd* age of enzymes using the age of the most ancient domain). (B) Comparison of clustering coefficient and average path length of the subnetwork one-mode network to that of an Erdős–Rényi network. Small-world coefficient decreases with the passage of time (*nd* age of enzymes using the age of the most ancient domain).
(TIF)

S10 Fig Matrix representation of subnetwork one-mode graphs by evolutionary age (*nd* age of enzymes using the age of the most ancient domain). Rows represent nodes (subnetworks) with each cell indicating the number of enzymes (edges) per subnetwork in each *nd* interval.
(TIF)

S11 Fig A reduced representation of the subnetwork-enzyme bipartite network at *nd* = 1.0 (*nd* age of enzymes using the age of the most ancient domain). It shows major vertices in metabolic subnetwork one-mode graph. The edge connectivity represents enzyme sharing with greyscale values indicating the number of enzymes among the subnetworks. A full description of KEGG subnetwork labels can be found in S2 Table.
(TIF)

S12 Fig Number of nodes in each network type at 0.1 *nd* intervals (*nd* age of enzymes using the age of the most ancient domain). (TIF)

S13 Fig Log-log plots for the bipartite network derived from subnetwork-enzyme connections at 0.1 *nd* interval (*nd* age of enzymes using the age of the most ancient domain). (TIF)

S14 Fig Full subnetwork one-mode projection (*nd* = 1.0) of the reduced representation of Fig 10. The network projection shows nodes (subnetworks) colored according to mesonetworks they belong to. The nodes are connected to each other through links based on sharing of enzymes. Link widths are proportional to the number of enzymes shared among the subnetworks. A full description of KEGG subnetwork labels can be found in S2 Table and S3 Table.
(TIF)

S1 Table Number of subnetworks associated with enzymes in KEGG and MANET expressed per mesonetwork. (XLSX)

S2 Table Painting efficiency of metabolic MANET compared to total number of nodes in KEGG metabolic pathways. (XLSX)

S3 Table Pathway names with their associated map numbers. (XLSX)

S4 Table EC name revisions for superkingdom annotations. EC numbers have been revised after the creation of MANET. This table includes changes after February, 2014 and before October, 2018. In order to conform to the EC numbers associated with the maps available on MANET, older EC numbers have been used in analysis. However, the superkingdom annotations used are derived from the revised EC numbers, if there was a clear consensus, in case of multiple EC annotations after revision.
(XLSX)

S5 Table Parameters for the power law fitting function in R for the bipartite network corresponding to the ancestral age networks at different *nd* values (*nd* age of enzymes using the age of the most ancient domain). alpha: exponent for the fitted power law distribution, xmin: lower bound for the power law fitting, logLik: log-likelihood of fitted parameters, KS.stat: test statistic for the Kolmogorov Smirnov test between fitted and sample distribution and KS.p: p-value for the Kolmogorov Smirnov test between fitted and sample distribution.
(XLSX)

S6 Table Results of the Bartels’ test for randomness performed on each type of network at each time-point (0.1 *nd* interval; *nd* age of enzymes using the age of the most ancient domain). The null hypothesis is that the underlying data has been drawn from a random distribution. p-values less than 0.05 indicate that the null hypothesis is rejected.
(XLSX)

S7 Table Network centrality metrics for the enzyme one-mode network extracted from the subnetwork-enzyme bipartite network by *nd* Values (*nd* age of enzymes using the age of the most ancient domain). (XLSX)
