# Supplementary figures and images for: MANET 3.0: Hierarchy and modularity in evolving metabolic networks
Source: PLoS One. 2019 Oct 24;14(10):e0224201. doi: 10.1371/journal.pone.0224201 (PMC6812854; doi:10.1371/journal.pone.0224201)

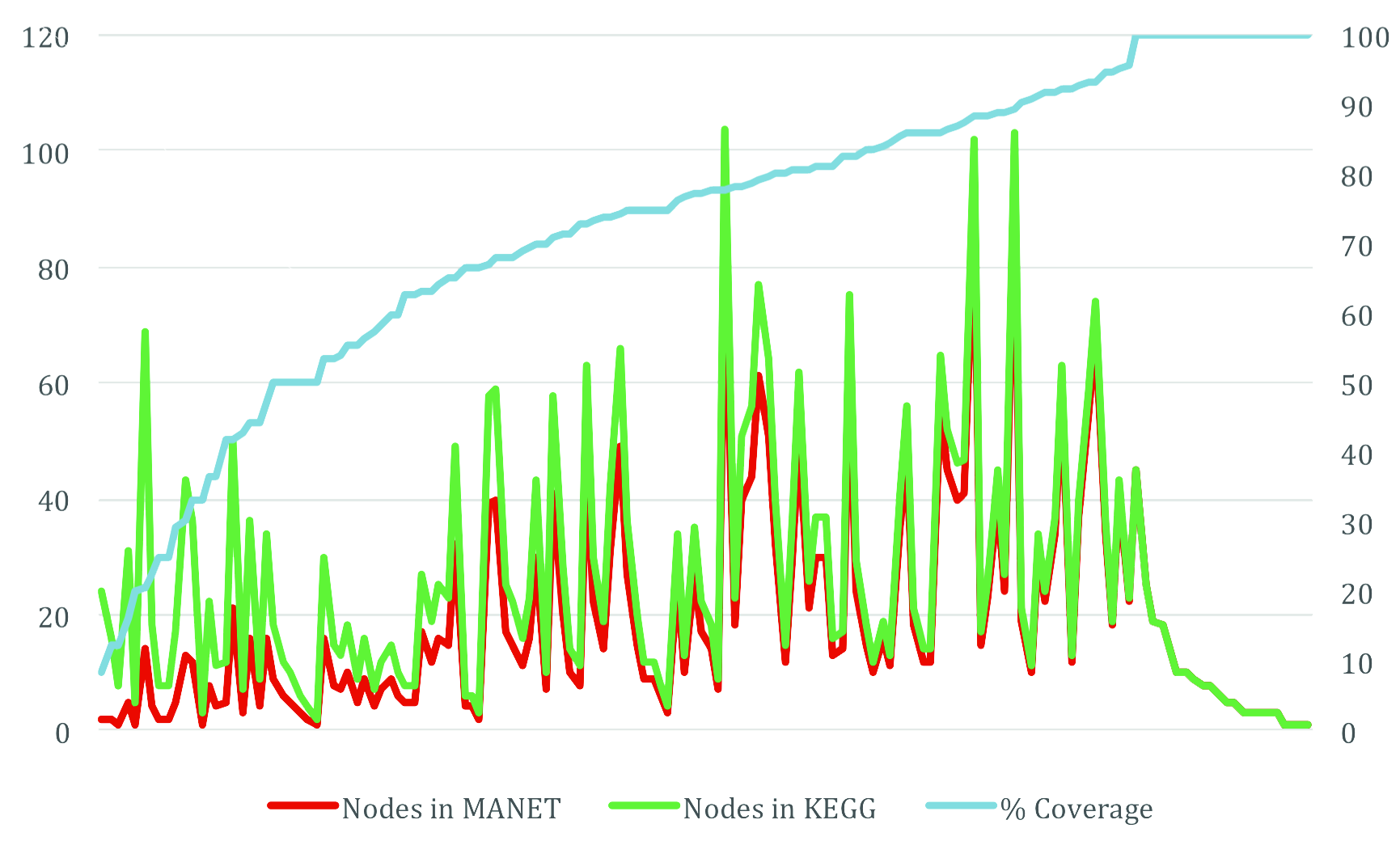

Supplement: S1 Fig — The x-axis represents the subnetwork number, while the y-axis (left) denotes number of enzymes per subnetwork. The y-axis (right) indicates percentage trend of the coverage. (TIF) [file pone.0224201.s001.tif]

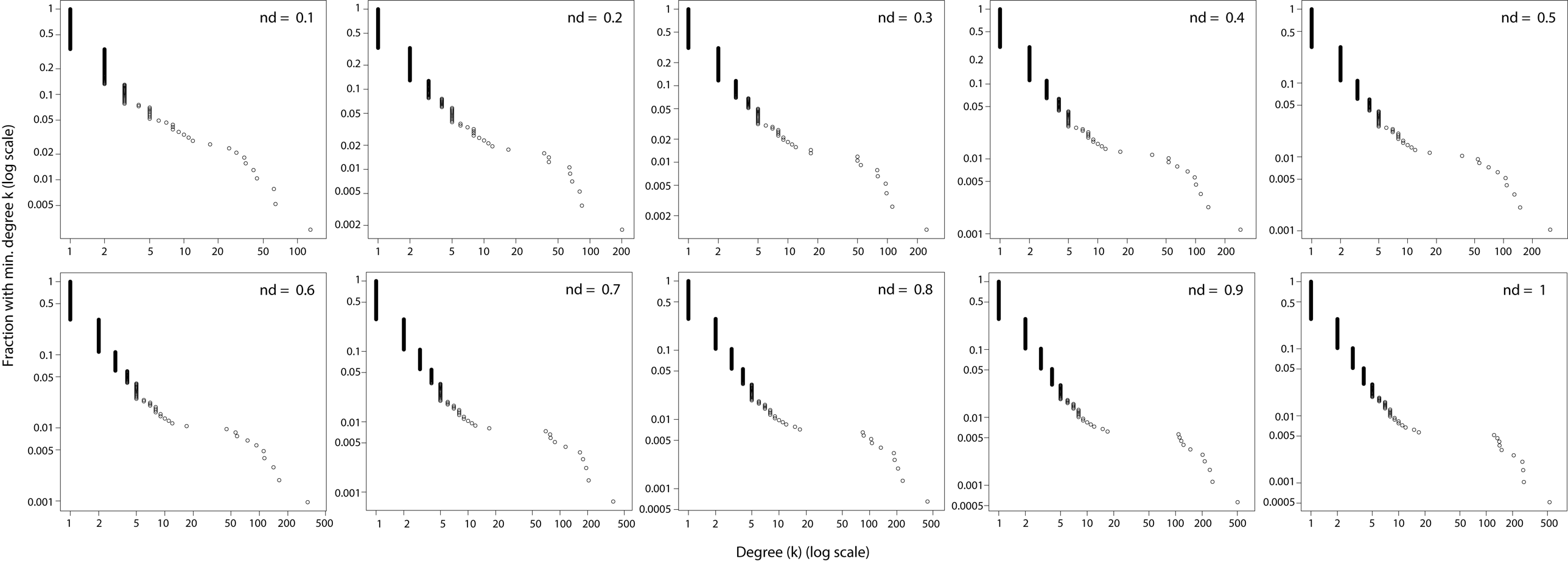

Supplement: S2 Fig — (TIF) [file pone.0224201.s002.tif]

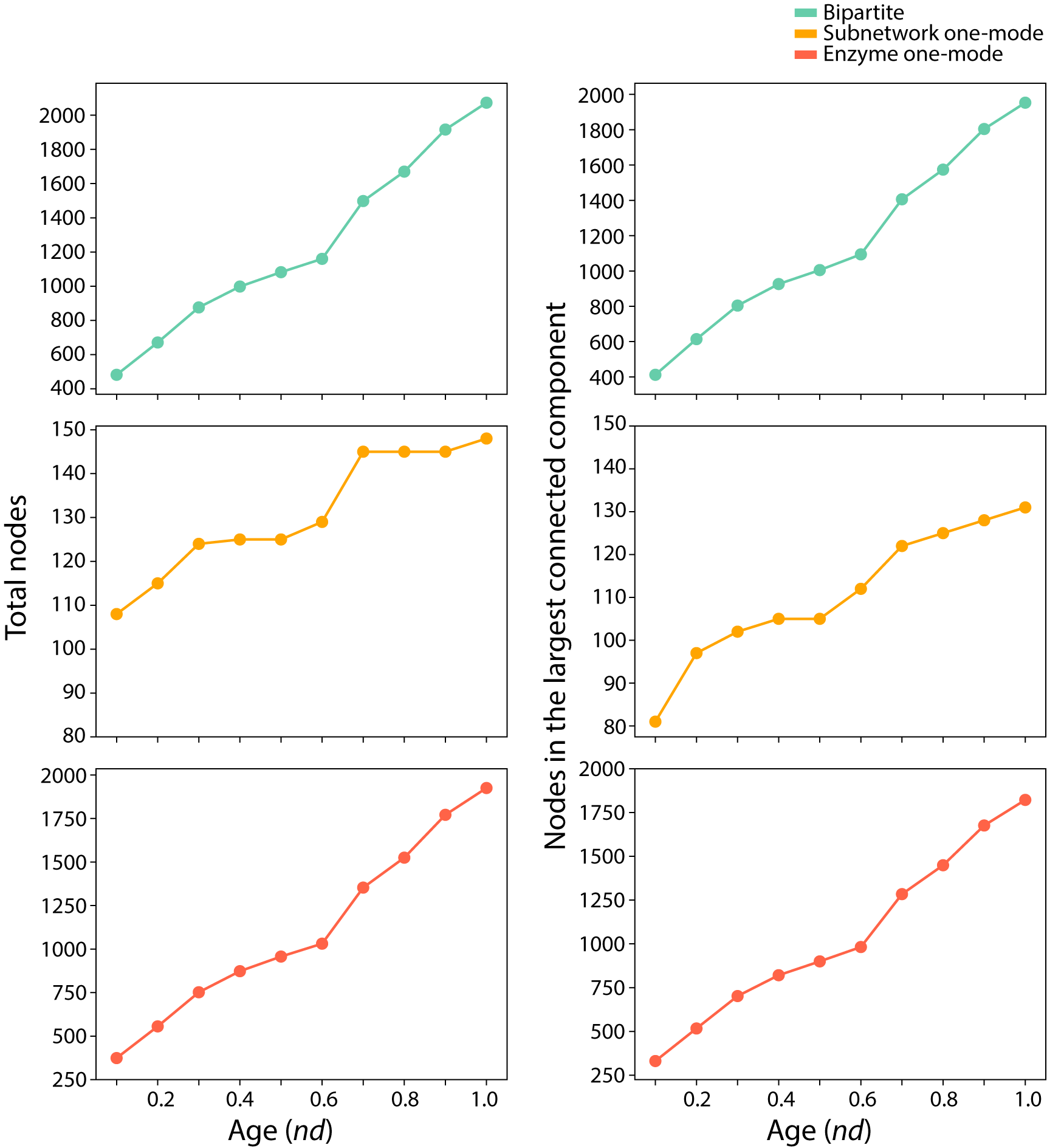

Supplement: S3 Fig — (TIF) [file pone.0224201.s003.tif]

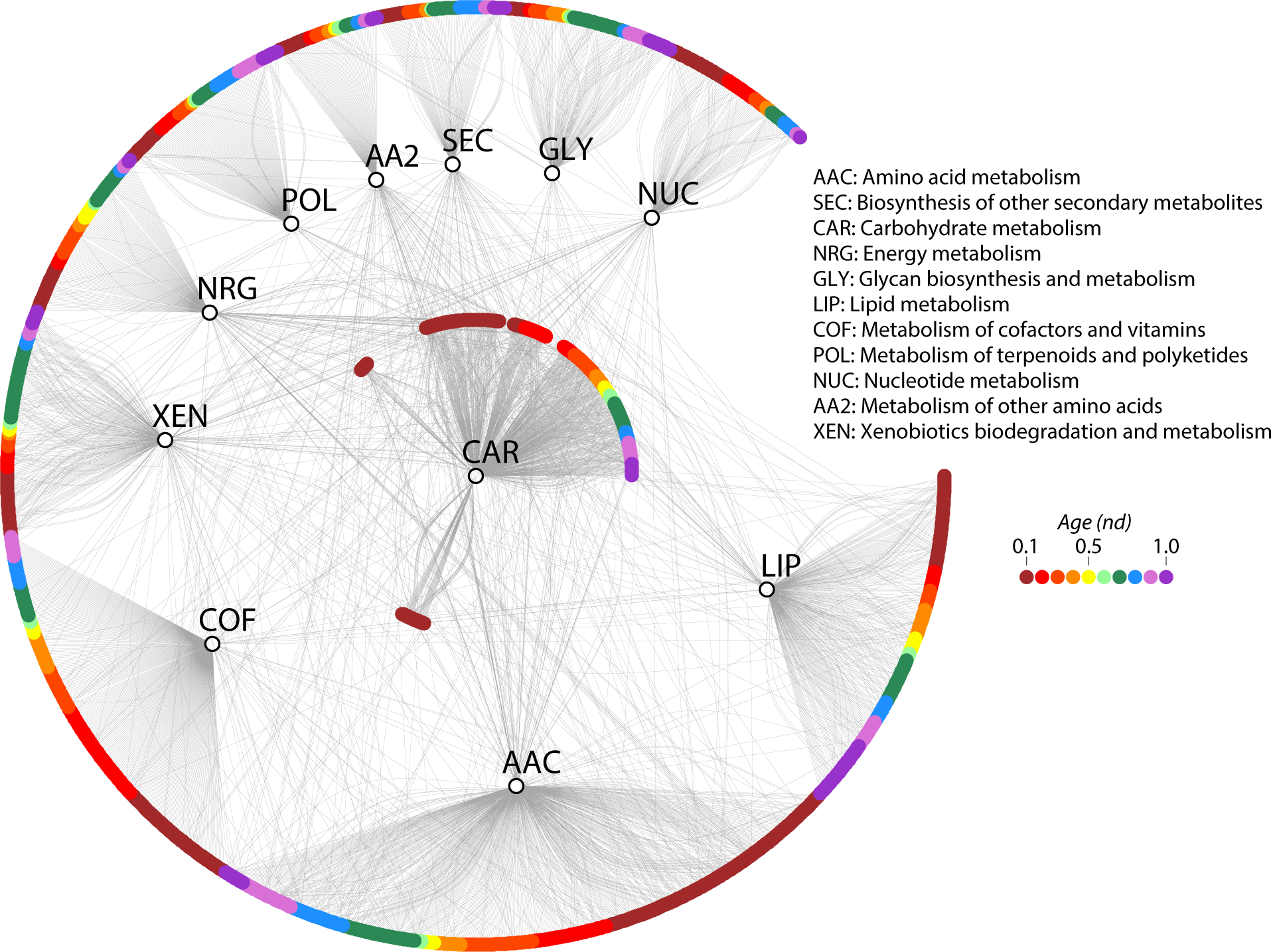

Supplement: S4 Fig — Mesonetwork are shown as open circles while colored nodes denote enzymes. (TIF) [file pone.0224201.s004.tif]

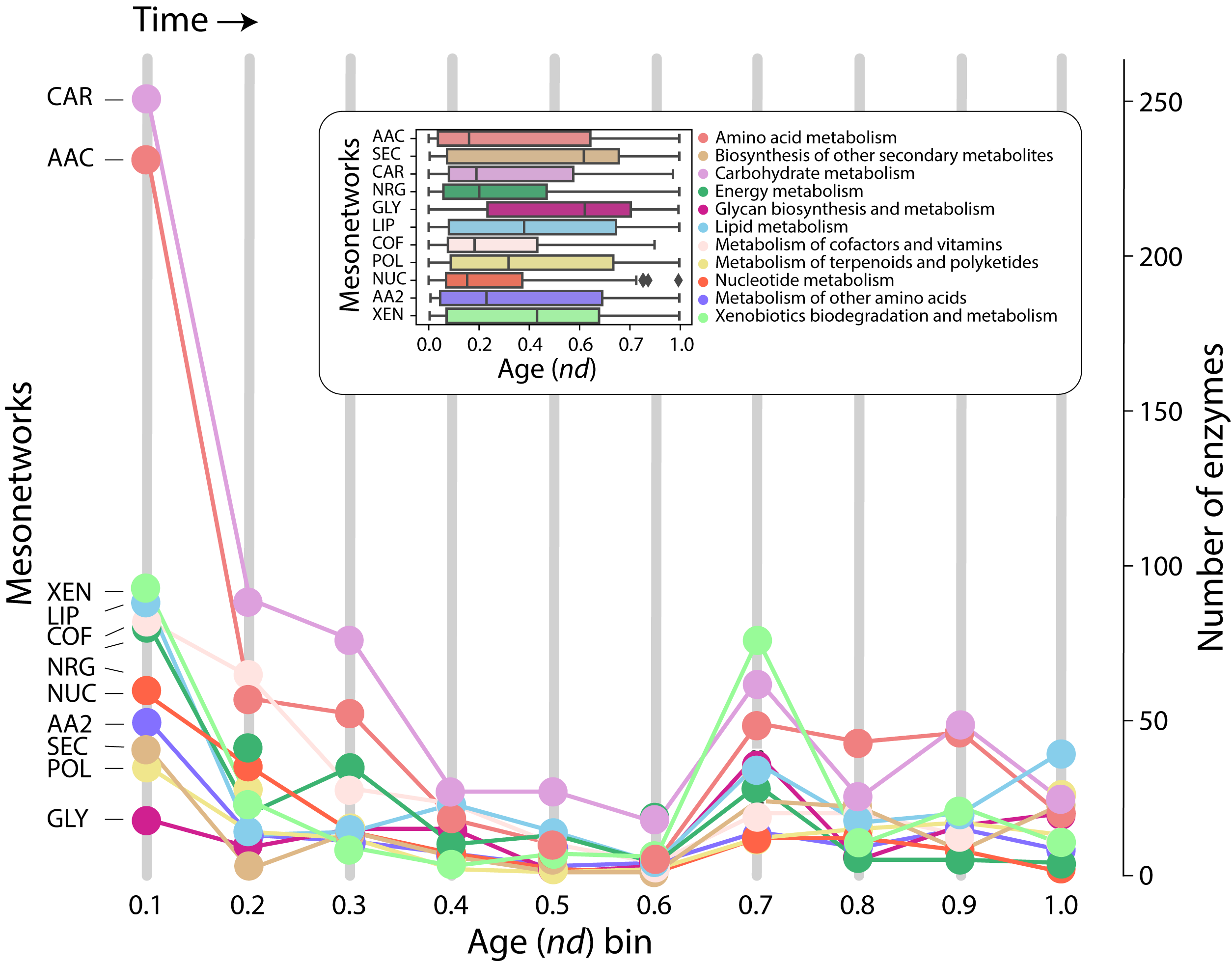

Supplement: S5 Fig — Eras are defined as nd bins of ages; the first nd bin includes enzymes appearing between nd = 0 and nd = 0.1. The inset describes the distribution of enzymes along the evolutionary timeline. (TIF) [file pone.0224201.s005.tif]

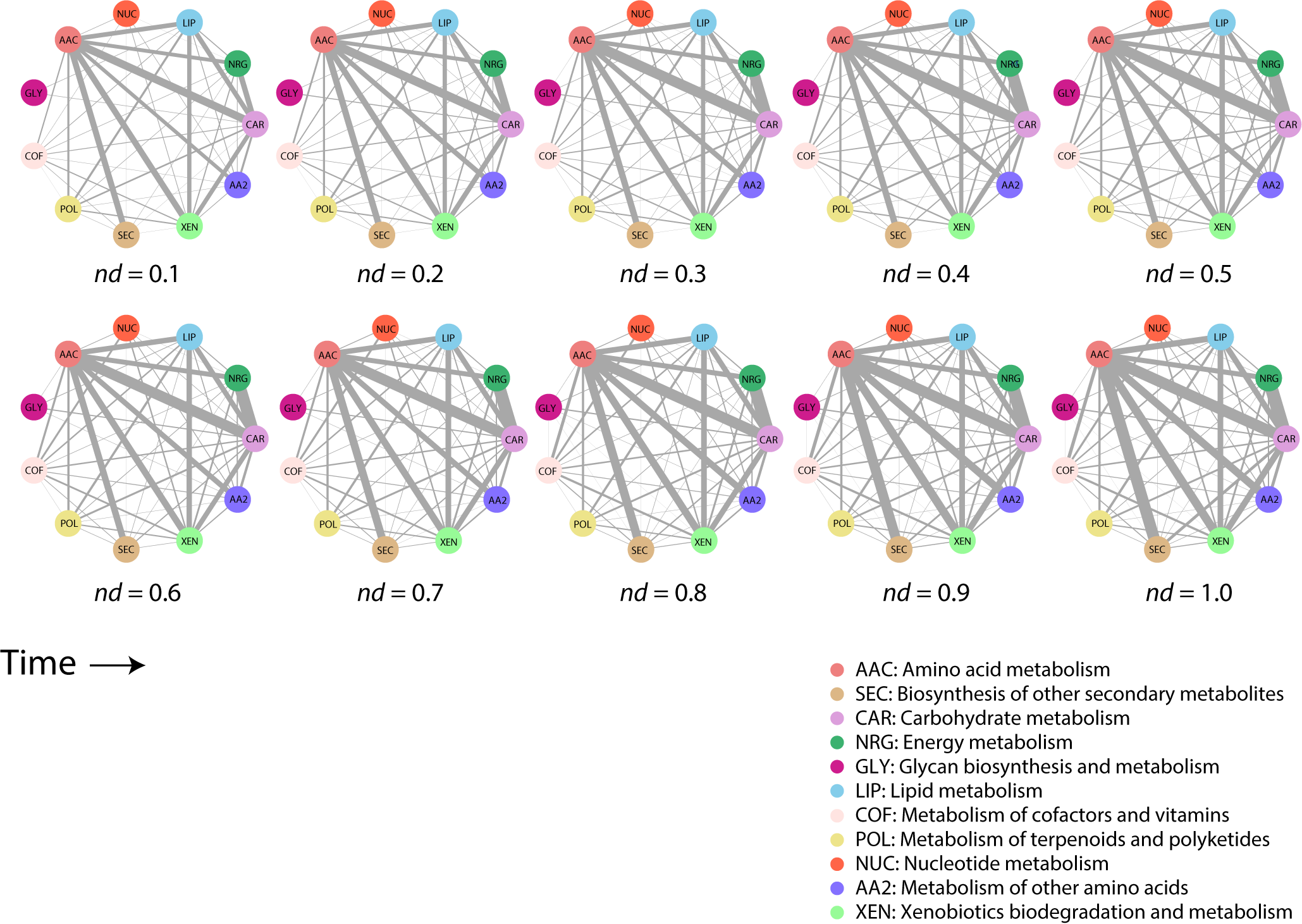

Supplement: S6 Fig — Mesonetworks are represented by vertices while edge thickness shows the number of enzymes shared. AAC, Amino acid metabolism; SEC, Biosynthesis of other secondary metabolites; CAR, Carbohydrate metabolism; NRG, Energy metabolism; GLY, Glycan biosynthesis and metabolism; LIP, Lipid metabolism; COF, Metabolism of cofactors and vitamins; POL, Metabolism of terpenoids and polyketides; NUC, Nucleotide metabolism; AA2, Metabolism of other amino acids; XEN, Xenobiotics biodegradation and metabolism. (TIF) [file pone.0224201.s006.tif]

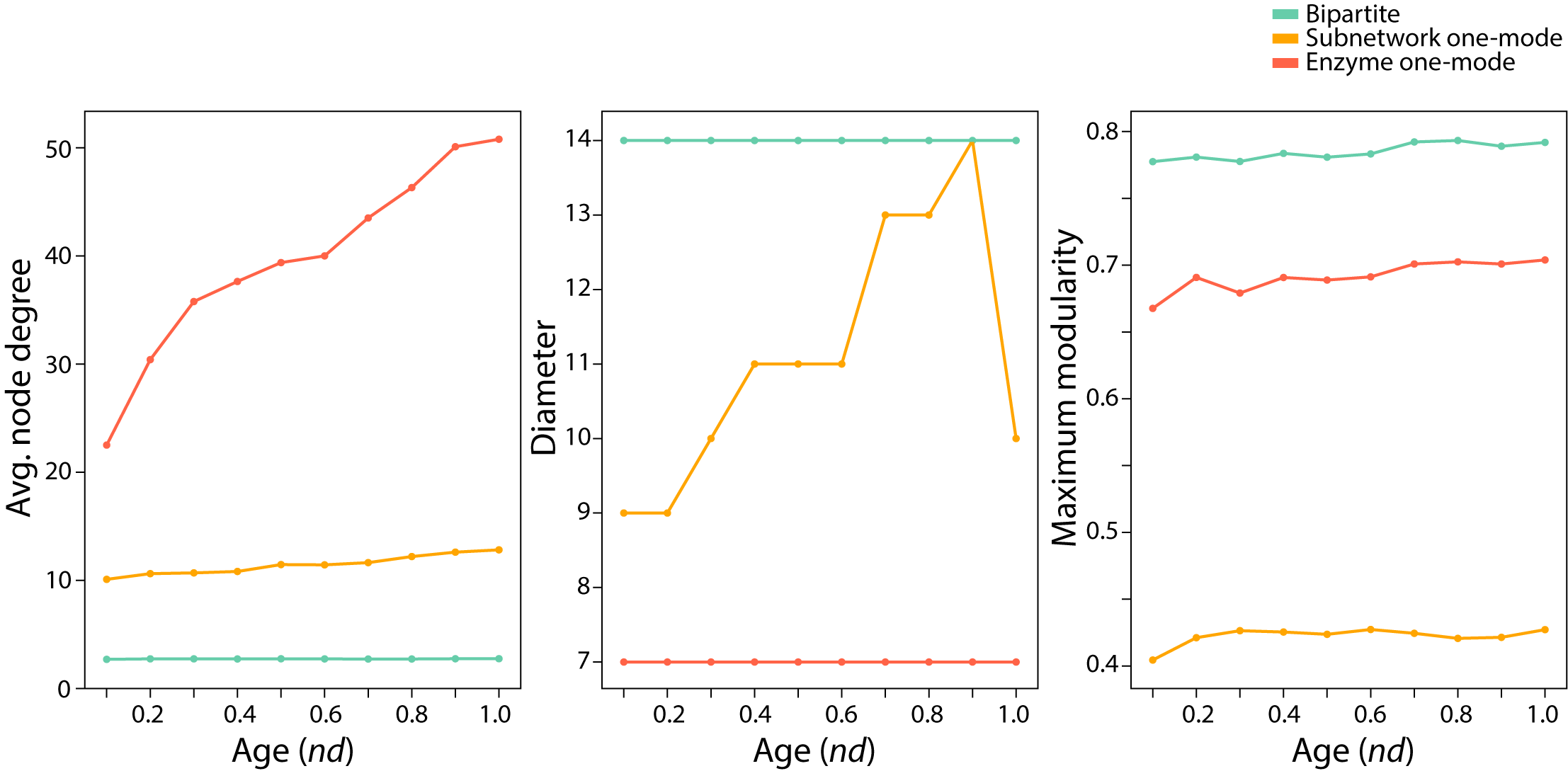

Supplement: S7 Fig — Network sizes (total number of nodes and nodes in the largest connected component) are given in S12 Fig. (TIF) [file pone.0224201.s007.tif]

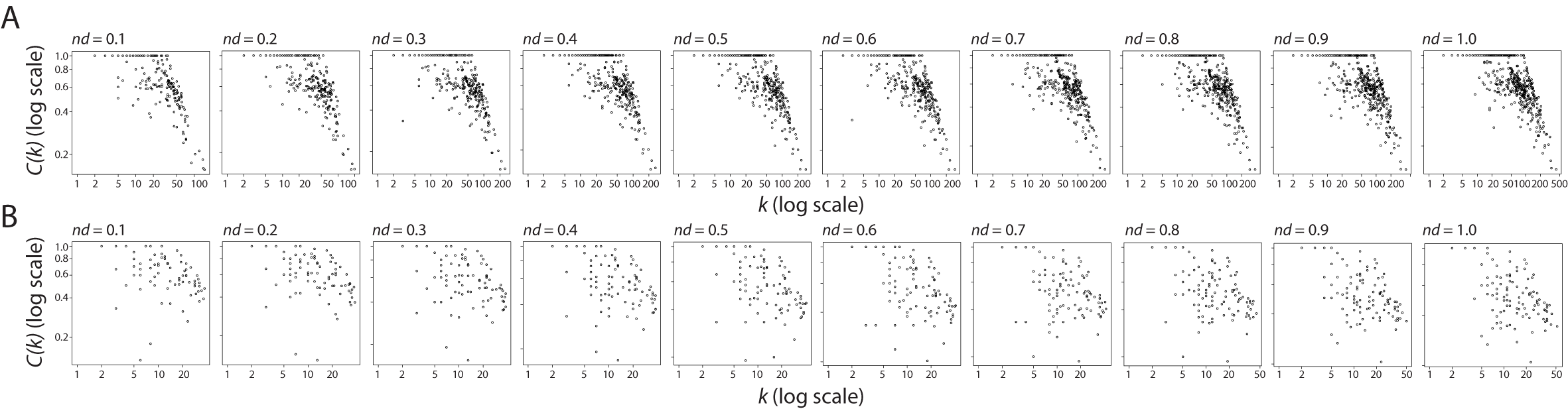

Supplement: S8 Fig — (TIF) [file pone.0224201.s008.tif]

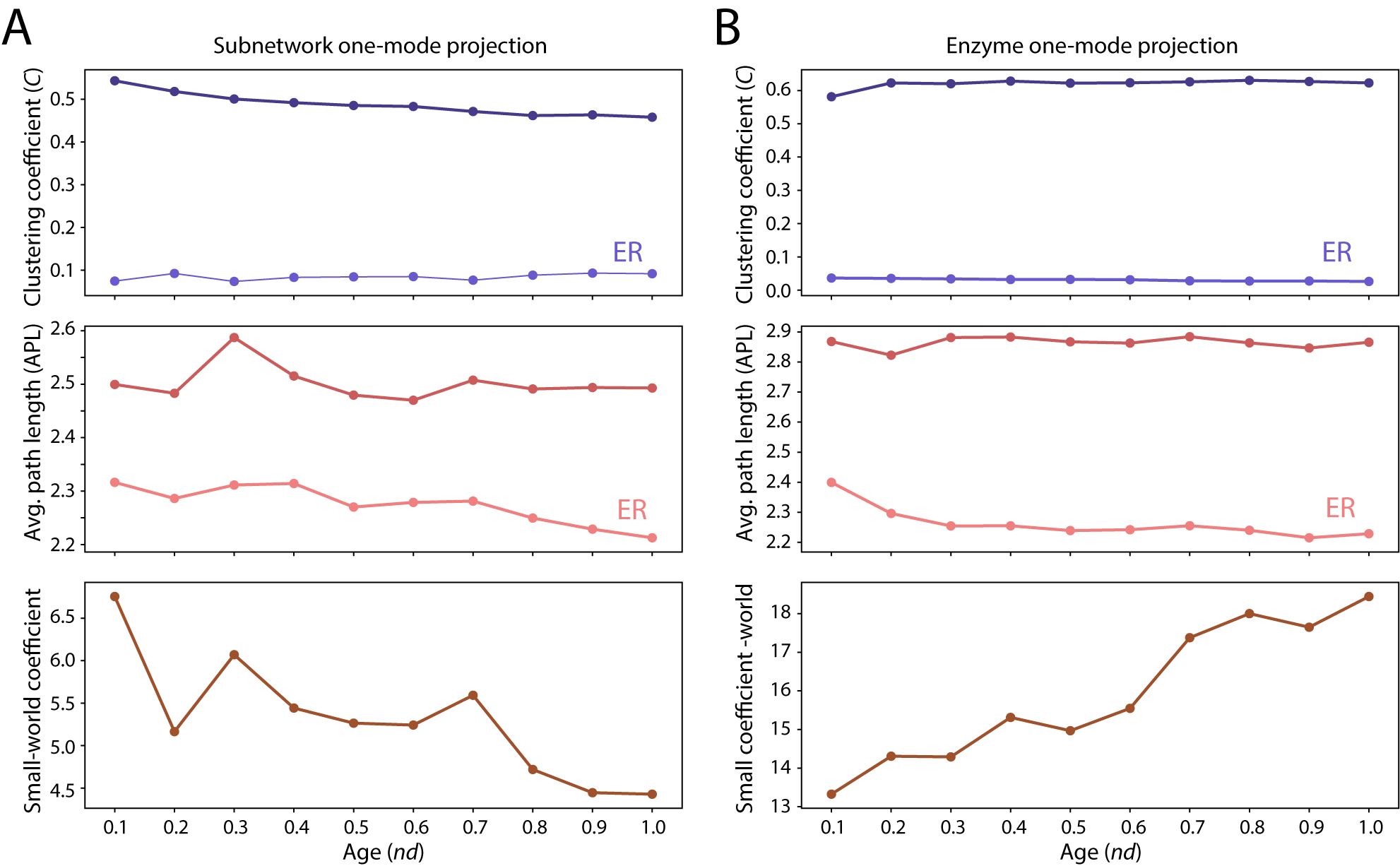

Supplement: S9 Fig — (A) Comparison of clustering coefficient and average path length of the enzyme one-mode network to that of an Erdős–Rényi network. The resulting small-world coefficient seems to increase along the evolutionary timeline (nd age of enzymes using the age of the most ancient domain). (B) Comparison of clustering coefficient and average path length of the subnetwork one-mode network to that of an Erdős–Rényi network. Small-world coefficient decreases with the passage of time (nd age of enzymes using the age of the most ancient domain). (TIF) [file pone.0224201.s009.tif]

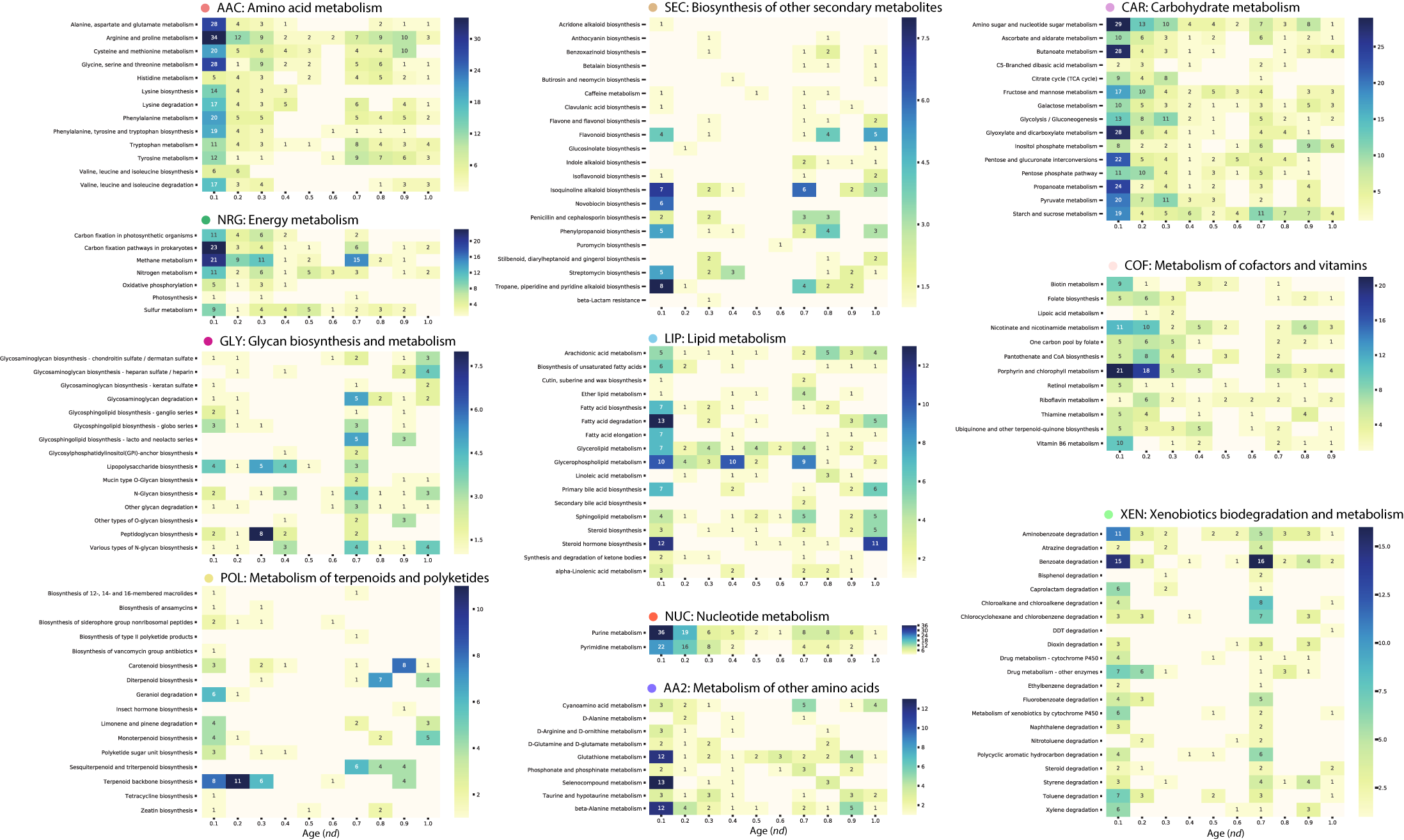

Supplement: S10 Fig — Rows represent nodes (subnetworks) with each cell indicating the number of enzymes (edges) per subnetwork in each nd interval. (TIF) [file pone.0224201.s010.tif]

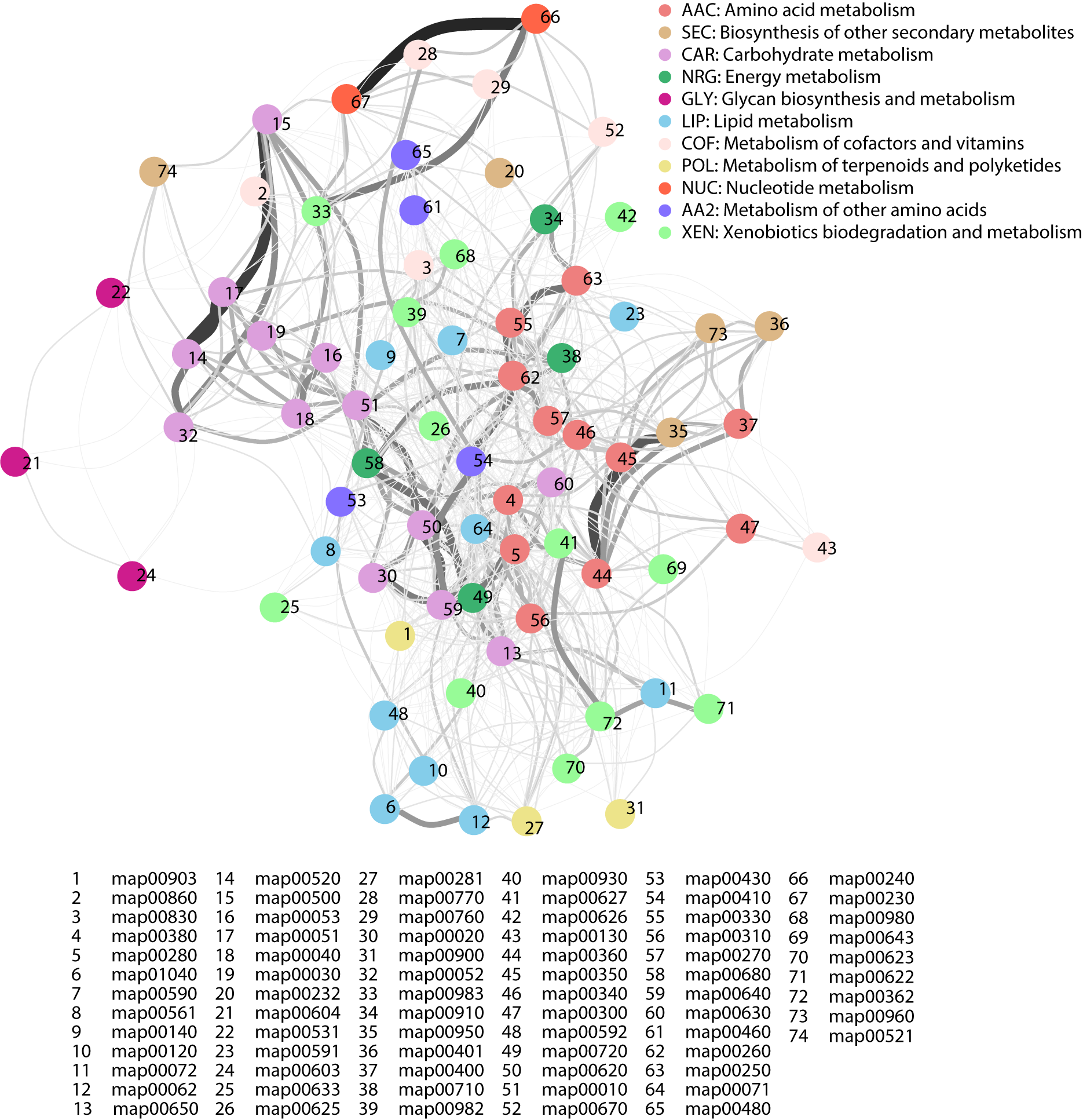

Supplement: S11 Fig — It shows major vertices in metabolic subnetwork one-mode graph. The edge connectivity represents enzyme sharing with greyscale values indicating the number of enzymes among the subnetworks. A full description of KEGG subnetwork labels can be found in S2 Table. (TIF) [file pone.0224201.s011.tif]

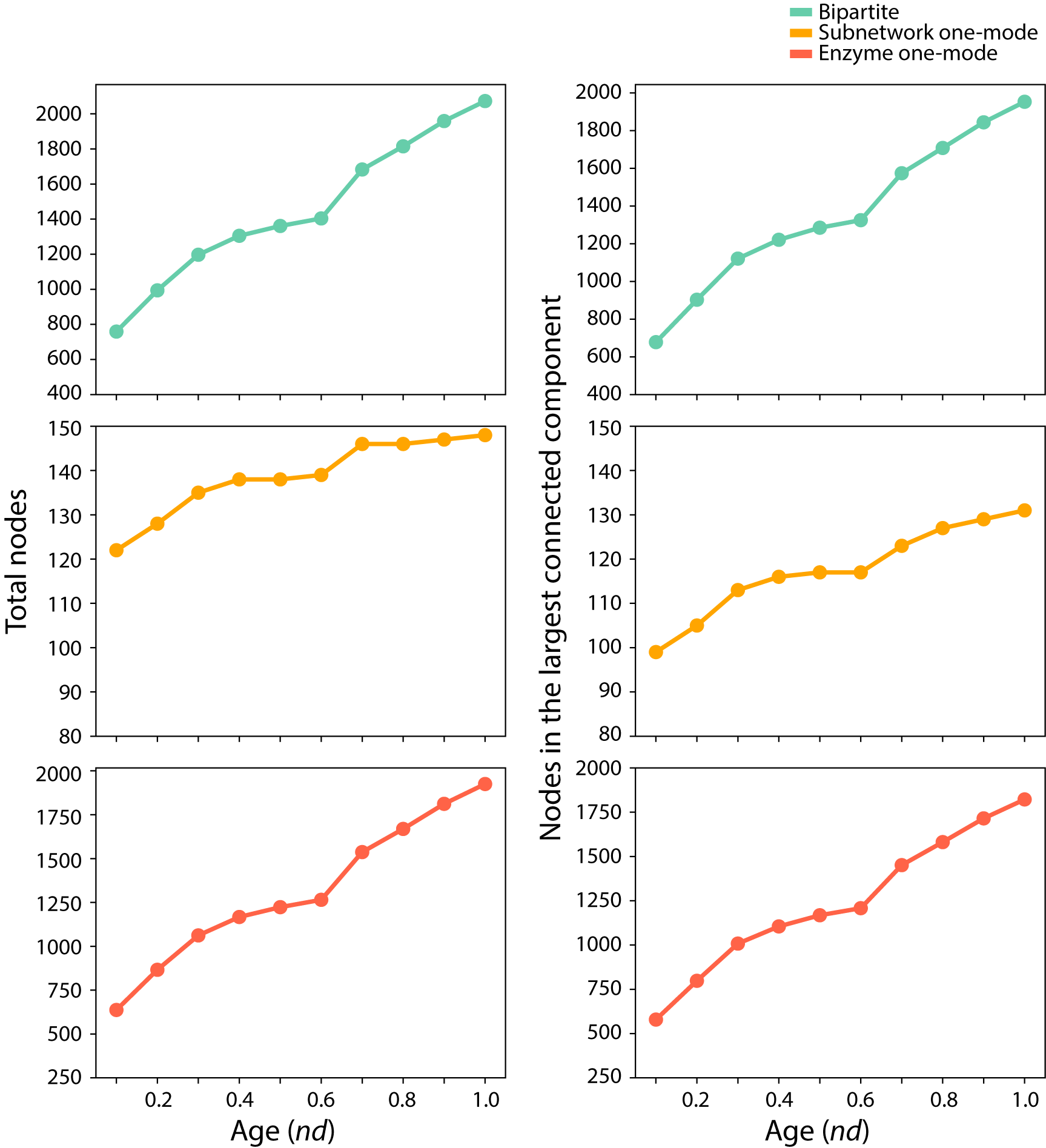

Supplement: S12 Fig — (TIF) [file pone.0224201.s012.tif]

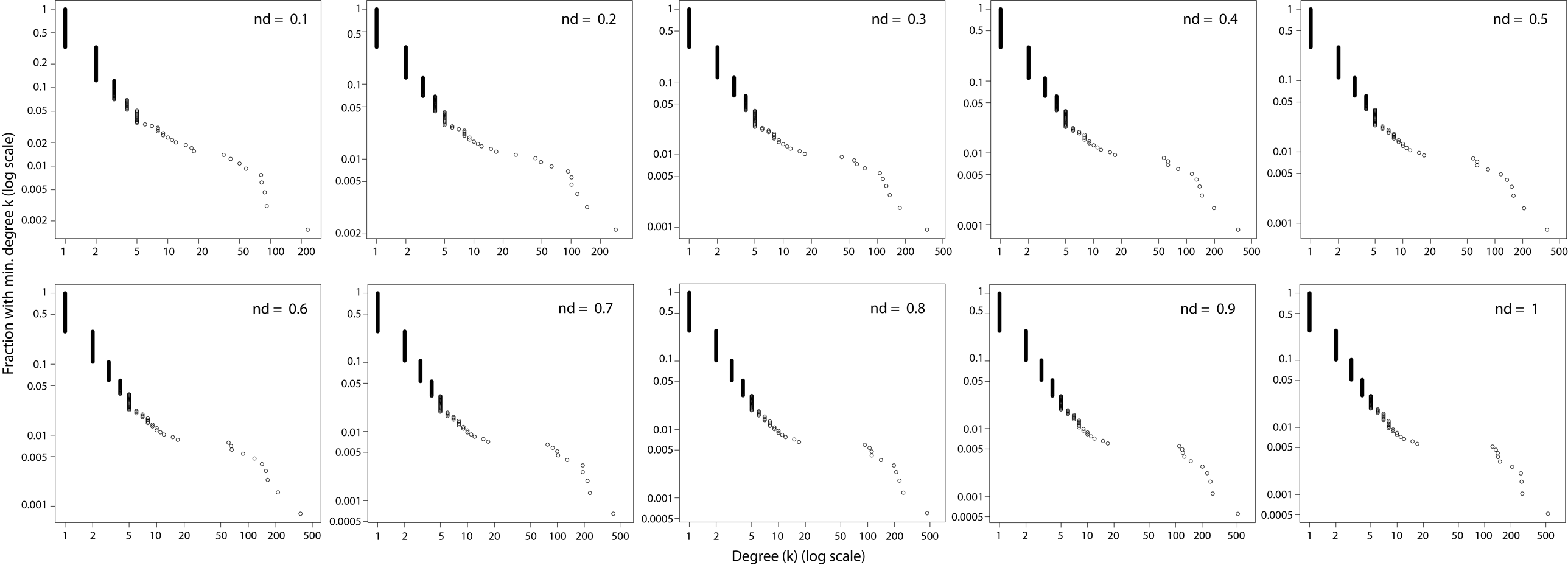

Supplement: S13 Fig — (TIF) [file pone.0224201.s013.tif]

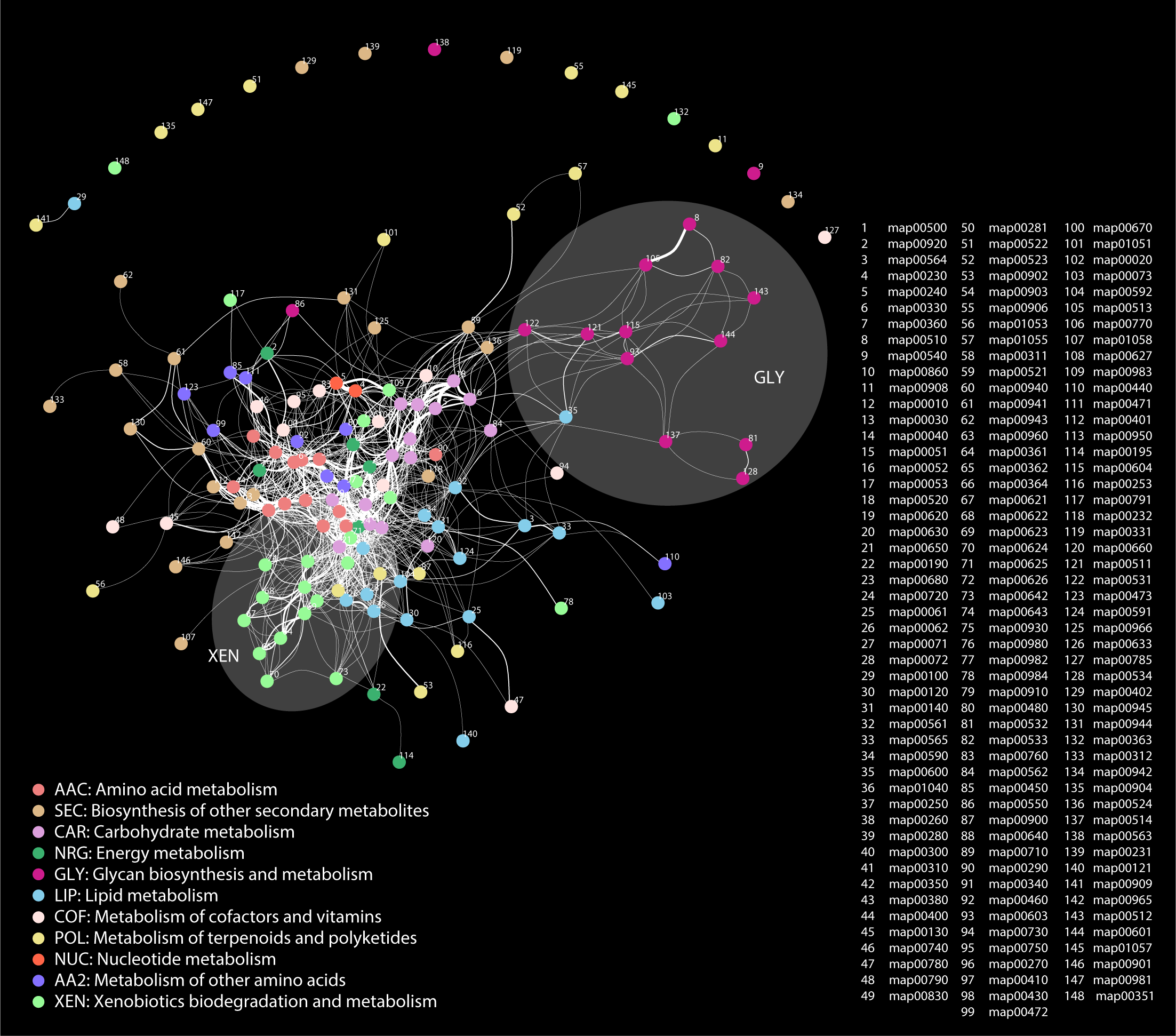

Supplement: S14 Fig — The network projection shows nodes (subnetworks) colored according to mesonetworks they belong to. The nodes are connected to each other through links based on sharing of enzymes. Link widths are proportional to the number of enzymes shared among the subnetworks. A full description of KEGG subnetwork labels can be found in S2 Table and S3 Table. (TIF) [file pone.0224201.s014.tif]
